# Semaphorin-3A regulates liver sinusoidal endothelial cell porosity and promotes hepatic steatosis

**DOI:** 10.1038/s44161-024-00487-z

**Published:** 2024-06-14

**Authors:** Daniel Eberhard, Sydney Balkenhol, Andrea Köster, Paula Follert, Eric Upschulte, Philipp Ostermann, Philip Kirschner, Celina Uhlemeyer, Iannis Charnay, Christina Preuss, Sandra Trenkamp, Bengt-Frederik Belgardt, Timo Dickscheid, Irene Esposito, Michael Roden, Eckhard Lammert

**Affiliations:** 1 Heinrich Heine University Düsseldorf, Faculty of Mathematics and Natural Sciences, Institute of Metabolic Physiology, Düsseldorf, Germany; 2 Institute for Vascular and Islet Cell Biology, German Diabetes Center (DDZ), Leibniz Center for Diabetes Research at Heinrich Heine University, Düsseldorf, Germany; 3 German Center for Diabetes Research (DZD), Neuherberg, Germany; 4 Cécile & Oskar Vogt Institute of Brain Research, Medical Faculty and University Hospital Düsseldorf, Düsseldorf, Germany; 5 Institute of Neuroscience and Medicine (INM-1), Research Center Jülich, Jülich, Germany; 6 Helmholtz AI, Research Center Jülich, Jülich, Germany; 7 Institute for Clinical Diabetology, German Diabetes Center, Leibniz Center for Diabetes Research at Heinrich Heine University, Düsseldorf, Germany; 8 Heinrich Heine University Düsseldorf, Faculty of Mathematics and Natural Sciences, Institute of Computer Science, Düsseldorf, Germany; 9 Institute of Pathology, Medical Faculty and University Hospital Düsseldorf, Heinrich Heine University, Düsseldorf, Germany; 10 Division of Endocrinology and Diabetology, Medical Faculty and University Hospital Düsseldorf, Heinrich Heine University, Düsseldorf, Germany

**Keywords:** Obesity, Liver diseases

## Abstract

Prevalence of metabolic dysfunction-associated steatotic liver disease (MASLD), formerly known as non-alcoholic fatty liver disease, increases worldwide and associates with type 2 diabetes and other cardiometabolic diseases. Here we demonstrate that *Sema3a* is elevated in liver sinusoidal endothelial cells of animal models for obesity, type 2 diabetes and MASLD. In primary human liver sinusoidal endothelial cells, saturated fatty acids induce expression of *SEMA3A*, and loss of a single allele is sufficient to reduce hepatic fat content in diet-induced obese mice. We show that semaphorin-3A regulates the number of fenestrae through a signaling cascade that involves neuropilin-1 and phosphorylation of cofilin-1 by LIM domain kinase 1. Finally, inducible vascular deletion of *Sema3a* in adult diet-induced obese mice reduces hepatic fat content and elevates very low-density lipoprotein secretion. Thus, we identified a molecular pathway linking hyperlipidemia to microvascular defenestration and early development of MASLD.

## Abstract (web summary)

Eberhard et al. show that SEMA3A regulates liver sinusoidal endothelial cell fenestrations by signaling through NRP1 and LIMK1, revealing a pathway that connects hyperlipidemia to the development of steatotic liver disease.

## Main

The liver is a key regulator of lipid metabolism. It receives blood-borne free fatty acids (FFAs), lipoproteins and carbohydrates and utilizes these to produce new triglycerides (TGs), which are packaged and secreted into the bloodstream as very low-density lipoproteins (VLDL)^1^. Previous data suggest that the exchange of macromolecules between the blood and hepatocytes is facilitated by highly permeable liver sinusoidal endothelial cells (LSECs), which separate the sinusoidal vascular lumen from the microvilli of the hepatocytes^2–5^. LSECs typically harbor 50–300 nm-sized fenestrae or pores, which are organized in sieve plates^2,3^. They are thought to allow a free passage of virtually all macromolecules as well as lipoproteins and their remnants, except for chylomicrons, which are too large^6^. The number of fenestrae and their diameter in LSECs is dynamic, thought to be adapted to physiologic needs^7^ and can (at least in vitro) be changed within minutes^8^.

The porosity of LSECs is reduced in liver tissue of aged mice and rats showing age-related capillarization of sinusoids, which is called ‘pseudocapillarization’ and is characterized by LSEC thickening, basement membrane formation and defenestration (loss of fenestrae)^9,10^. Most notably, pseudocapillarization and defenestration are observed in chronic liver diseases, including MASLD, previously known as ‘non-alcoholic fatty liver disease’ (NAFLD), and may precede more severe stages of MASLD, including fibrosis and inflammation, culminating in metabolic dysfunction-associated steatohepatitis (MASH), previously known as ‘non-alcoholic steatohepatitis’ (NASH)^11–13^. For example, in mice fed with a choline-deficient l-amino acid-defined diet, LSEC porosity (the ratio of fenestrae area to total cell area) declines before severe steatosis with ‘ballooning hepatocytes’^14^. Moreover, a rise in the blood concentration of palmitic acid correlates with a reduced porosity of LSECs in high-fat diet (HFD)-fed mice^15^. Information about LSEC porosity and defenestration in individuals with MASLD is still scarce, even though a recent study reported less defenestration in liver biopsies from human individuals with MASH compared to those with MASLD, but without MASH^16^, indicating that defenestration preferentially takes place during an early stage of MASLD. It can be speculated that defenestration of LSEC reduces the export of VLDL from the liver (possibly contributing to hepatic steatosis) and prevents hepatic removal of chylomicron remnants from the bloodstream (possibly contributing to hyperlipidemia)^5,17^, thus triggering early development of MASLD; however, the molecular basis of LSEC defenestration and its contribution to MASLD, including genetic triggers of defenestration, are largely unknown^11^.

Class 3 semaphorins (SEMA3A-G) play a major role in various biological processes and human disorders, including neural and cardiovascular development, adipogenesis, adipose tissue function, hypothalamus regulation of obesity, inflammation and energy balance^18–20^. SEMA3A is a secreted protein and binds to neuropilin-1 (NRP1) or neuropilin-2 (NRP2), whereas NRP forms a holoreceptor complex with plexins present on both vascular and lymphatic endothelial cells^21,22^. SEMA3A counteracts parts of the signaling pathway of vascular endothelial growth factor-A (VEGF-A), which is a key driver of LSEC fenestration, as disruption of VEGF-A signaling causes defenestration of LSECs as well as defenestration of other endothelial cell types in vivo^23–25^. Notably, plasma concentrations of SEMA3A have been reported to be elevated in individuals with obesity and type 2 diabetes (T2D)^26^ as well as in individuals with MASLD^27^, pointing to a potential relevance of SEMA3A in human obesity and T2D. In the cirrhotic rat liver, *Sema3a* is upregulated in LSECs^28^. SEMA3A has also been reported to modulate actin filaments in several cell types and cause disorganization of filamentous-actin (F-actin) stress fibers in endothelial cells^29^. Therefore, SEMA3A may impact LSEC fenestration in analogy to exogenous actin-binding substances that alter fenestration^3,4^. Here we investigated the hypothesis that SEMA3A regulates LSEC porosity with a potential impact on intrahepatic fat content.

## Results

### Higher *Sema3a* expression in hepatic steatosis

**Fig. 1:**
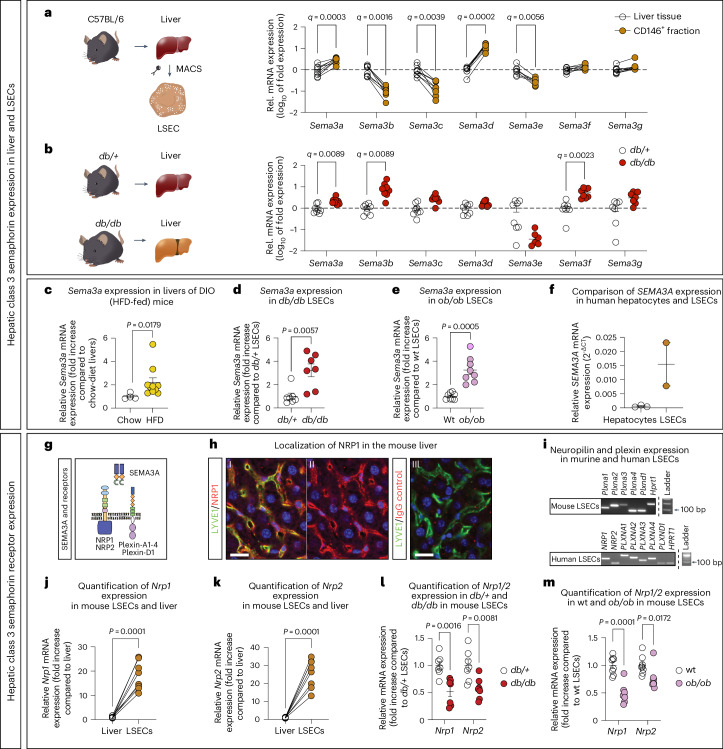
*Sema3a*/*SEMA3A* is expressed in LSECs and increased in mice with hepatic steatosis. **a**, *Sema3a-g* mRNA expression (log_10_ fold change) in LSECs compared to total liver of 14-week-old male C57BL/6 wild-type (wt) mice (*n* = 8, *n* = 7 for *Sema3e*). Lines indicate the same mouse. **b**, *Sema3a-g* mRNA expression (log_10_ fold change, log(0) values are not displayed) in liver tissue of 12-week-old *db*/*db* mice, compared to *db*/*+* controls (RT–qPCR; *n* = 8 each). A multiple two-tailed paired (**a**) or unpaired (**b**) Student’s *t*-test was used to discover significant effects^84^. Discoveries are indicated by *q* values in **a**,**b**. **c**, *Sema3a* mRNA expression in liver tissue from standard chow-fed (*n* = 4, RT–qPCR) versus HFD-fed (*n* = 10) littermates. **d**, *Sema3a* mRNA expression of LSECs from 12-week-old male *db*/+ control versus *db/db* mice (*n* = 7 each). **e**, *Sema3a* mRNA expression of LSECs from 12-week-old male wt control versus *ob/ob* mice (*n* = 8 each). **f**, Relative *Sema3a* mRNA expression in hepatocytes and LSECs isolated from three and two human donors, respectively (RT–qPCR). **g**, Graphical overview of SEMA3A and its known receptors. **h**, Representative immunofluorescent staining for (I and II) neuropilin-1 (red, NRP1), (I and III) LYVE1 (green) and (III) goat IgG isotope control (red) of liver sections of C57BL/6 wt mice (*n* = 2 mice). Scale bars, 20 µm. **i**, Agarose gel with PCR products (RT–PCR) showing the expression of several SEMA3A receptors in primary mouse (*n* = 2 LSEC isolation) and human LSECs (male LSEC donor QC-12B15F11). Brightness and contrast have been adjusted to enhance visibility in **h**,**i**. **j**,**k**, *Nrp1* (**j)** and *Nrp2* (**k**) mRNA expression in LSECs compared to total liver from 14-week-old male C57BL/6 wt mice (*n* = 8 each). **l**,**m**, *Nrp1* and *Nrp2* mRNA expression in LSECs from *db/db* (**l**; *n* = 7) and *ob/ob* mice (**m**; *n* = 8) in comparison to controls. A two-tailed unequal variances *t*-test was used (**c**–**e**,**l**,**m**) and two-tailed paired *t*-test (**j**,**k**). Data are presented as mean ± s.e.m. CD146^+^ LSECs were isolated by MACS or FACS after MACS (**d**,**e**,**l**,**m**) to get an even higher purity of cells. Source data

**Extended Data Fig. 1:**
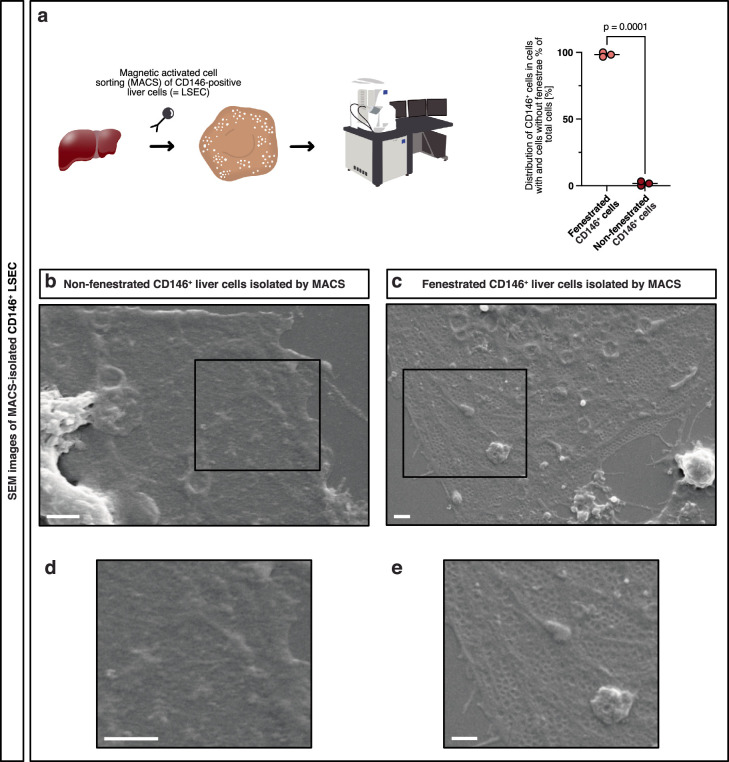
Efficient LSEC isolation from the mouse liver by MACS. **a**, After isolating CD146-positive liver cells (termed ‘LSEC’) by MACS, cells were allowed to grow for 4 h prior to SEM analysis. Example images are shown for MACS-isolated cells. **b**, Non-fenestrated CD146-positive liver cells (scale bar = 1 µm). **c**, Fenestrated CD146-positive liver cells (scale bar = 2 µm). **d**, **e**, Enlargement of regions indicated in (b) and (c) (scale bars = 2 µm). For statistical analysis, a two-tailed unequal variance *t*-test was performed (n = 3 independent LSEC isolations; for each isolation, 60 cells were randomly chosen and analyzed). Individual data points and mean ± SEM are presented. Source data

MASLD is associated with a lower LSEC porosity in mice^14,30^. To uncover a possible role of endothelial cell-derived class 3 semaphorins in MASLD, we first studied their expression in liver samples and isolated LSECs from wild-type C57BL/6 mice (Fig. 1a). To this end, we collected messenger RNA (mRNA) from total liver tissue and CD146-positive cells (a cell population largely consisting of mouse LSECs^31^) that were isolated from dispersed liver cells^32^. In the CD146-positive cell population, 98% of the cells were fenestrated and thus definitely LSECs (*P* = 0.0001; Extended Data Fig. 1). RT–qPCR analyses revealed that among the seven members of class 3 semaphorins, mainly *Sema3a* and *Sema3d* were enriched in mouse LSECs compared to total liver tissue (Fig. 1a and Supplementary Table 1).

Next, we investigated whether the expression of class 3 semaphorins was altered in animal models with hepatic steatosis^33^. Therefore, we first quantified class 3 semaphorin expression in liver samples from 12-week-old *db/db* mice, a commonly used mouse model for hepatic steatosis due to massive obesity and the development of T2D^33^. RT–qPCR analysis revealed higher expression of *Sema3a*, *Sema3b* and *Sema3f* in liver tissue from *db/db* mice versus *db*/+ control mice (Fig. 1b), which are normoglycemic, have normal body weight and lack steatosis^34^. Notably, these experiments revealed *Sema3a* as an LSEC-enriched class 3 semaphorin expressed to a higher extent in the liver of *db/db* versus *db*/+ mice (Fig. 1a,b). Likewise, *Sema3a* expression was elevated in the steatotic liver of diet-induced obese (DIO) mice fed with an HFD for 28 weeks compared to standard chow-fed littermate controls (Fig. 1c), further pointing to a possible role of *Sema3a* in the development of MASLD. To evaluate to which extent LSECs contribute to *Sema3a* upregulation in obese mice, we isolated CD146-positive cells from dissociated liver tissue by fluorescence-activated cell sorting (FACS) and quantified *Sema3a* expression by RT–qPCR in *db/db* mice as well as obese but non-diabetic *ob/ob* mice (Supplementary Information). We found that *Sema3a* expression was threefold higher in LSECs isolated from *db/db* and *ob/ob* mice compared to their respective controls (Fig. 1d,e). In analogy to mouse LSECs, *SEMA3A* was also expressed in primary human LSECs isolated from two human donors, whereas its expression was barely detectable in primary hepatocytes isolated from three human donors (Fig. 1f).

With regard to the SEMA3A receptors NRP1 and NRP2 (Fig. 1g), immunofluorescence staining and RT–qPCR confirmed their presence on mouse LSECs (Fig. 1h–k)^35^. We also observed stronger mRNA expression for *Nrp1* and *Nrp2* in mouse LSECs versus total mouse liver (Fig. 1j,k) and mRNA expression for the SEMA3A co-receptors plexin-A1-4 and plexin-D1 could be shown in both, primary mouse and human LSECs (Fig. 1i). Consistent with multiple mechanisms of NRP1 desensitization upon hyperstimulation^36,37^, we observed a reduction of *Nrp1* and *Nrp2* in *db/db* and *ob/ob* LSECs compared to their respective controls (Fig. 1l,m). In conclusion, mouse and human LSECs express *Sema3a/SEMA3A* and its expression is higher in LSECs from mice with hepatic steatosis compared to those without. Moreover, LSECs express mRNA of all known SEMA3A receptors, potentially enabling autocrine SEMA3A signaling in LSECs.

### Palmitic acid increases *SEMA3A* expression in human LSECs

**Fig. 2:**
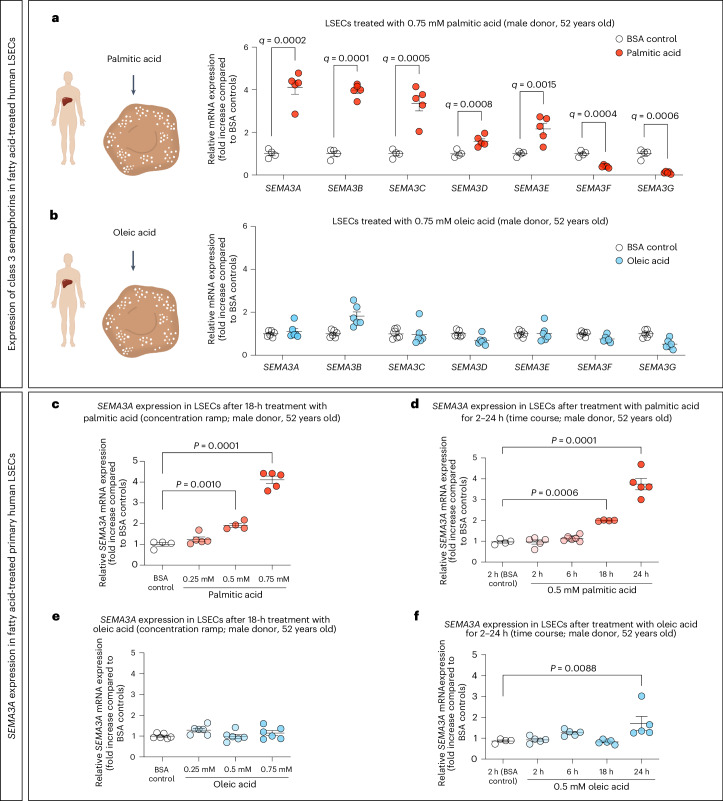
*SEMA3A* expression is upregulated in human LSECs after treatment with palmitic acid. **a**,**b**, *SEMA3A-G* mRNA expression in primary human LSECs (male donor QC-12B15F11) treated for 18 h with BSA control (*n* = 4 wells) versus 0.75 mM palmitic acid (*n* = 5 wells) (**a**) or oleic acid versus BSA controls (*n* = 6 wells each) (**b**). **c**–**f**, Expression of *SEMA3A* in primary human LSECs after treatment with BSA control (*n* = 4 palmitic acid-, *n* = 6 oleic acid-treated wells) versus 0.25 mM (*n* = 5, *n* = 6 wells), 0.5 mM (*n* = 4, *n* = 6 wells) and 0.75 mM (*n* = 5, *n* = 6 wells) BSA-bound palmitic acid (**c**) or oleic acid (**e**). Expression of *SEMA3A* in primary human LSECs treated with BSA controls (*n* = 4 wells each), 0.5 mM palmitic acid (**d**) or oleic acid (**f**) for 2 h (*n* = 5 wells each), 6 h (*n* = 6, *n* = 5 wells), 18 h (*n* = 4, *n* = 5 wells) and 24 h (*n* = 5 wells each). A multiple two-tailed unpaired *t*-test with a two-stage step-up method was used to discover outstanding effects^84^, as indicated by *q* values in **a**,**b**. A one-way ANOVA with Dunnett’s post hoc test was used to test for statistical significance in **c**–**f**. In all graphs individual data points and mean ± s.e.m. are presented. Source data

**Extended Data Fig. 2:**
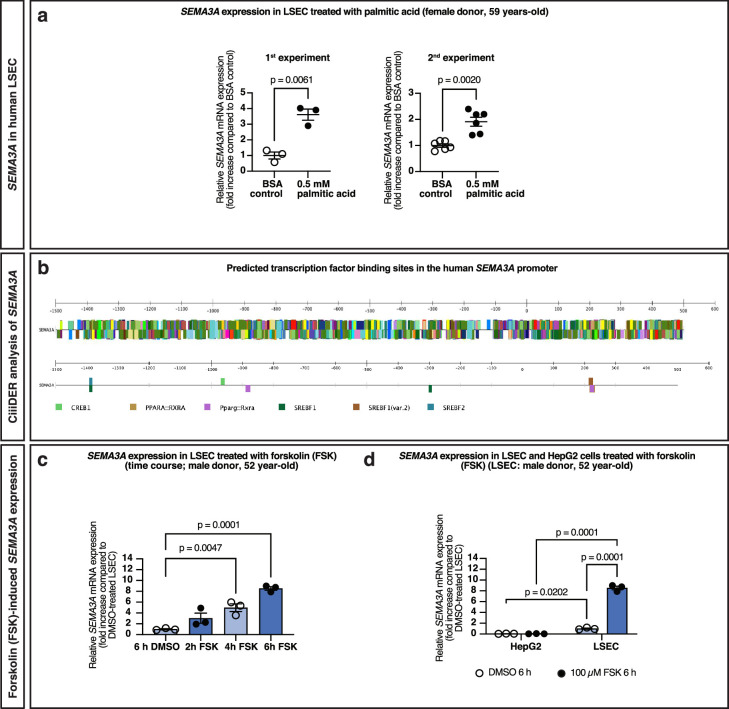
Upregulation of *SEMA3A* in primary human LSEC by palmitic acid and forskolin. **a**, *SEMA3A* mRNA expression in primary human LSEC (female donor; QC-29B15F09) treated for 24 h with 0.5 mM palmitic acid (n = 2 independent experiments, left n = 3, and right n = 6 wells per condition). **b**, Graphical overview of transcription factor (TF) binding sites predicted by CiiiDER (upper panel) and selected binding sites for CREB1, PPARs, and SREBFs (lower panel). **c**, *SEMA3A* mRNA expression in primary human LSEC (male donor; QC-12B15F11) treated with 100 µM forskolin (FSK) for 2, 4, and 6 h compared to DMSO-treated cells (n = 3 independent experiments). **d**, *SEMA3A* mRNA expression in HepG2 cells and primary human LSEC (male donor QC-12B15F11) after treatment with 100 µM FSK or DMSO for 6 h (n = 3 independent experiments). A two-tailed unequal variances *t*-test was used to test for statistical significance in (**a**). A one-way ANOVA with Dunnett´s *post hoc* test (**c**) and two-way ANOVA with Tukey´s *post hoc* test (**d**) were also used to test for statistical significance. In all graphs individual data points and mean ± SEM are presented. Source data

Circulating levels of palmitic acid, a saturated FFA, have been found to correlate with a lower fenestrae frequency and porosity in mouse LSECs^15^ and, if elevated, have been shown to induce both hepatic insulin resistance and steatosis in vitro and in vivo^38,39^. Therefore, we investigated whether expression of *SEMA3A* and other class 3 semaphorins were altered in primary human LSECs (male donor QC-12B15F11) after exposure to bovine serum albumin (BSA)-bound palmitic acid (Fig. 2), or BSA-bound oleic acid, a mono-unsaturated FFA with fewer deleterious effects than palmitic acid^40,41^. Notably, we found that all class 3 semaphorins were differentially expressed after treatment with 0.75 mM palmitic acid for 18 h (Fig. 2a), but not after exposure to 0.75 mM oleic acid (Fig. 2b), indicating that expression of this family of secreted factors is sensitive to the specific type of FFA at concentrations reported to be present in human plasma^42,43^. Further, *SEMA3A* was the most strongly upregulated class 3 semaphorin after palmitic acid treatment compared to the corresponding BSA control (Fig. 2a). In addition, treatment of primary human LSECs with this FFA resulted in a significant concentration- and time-dependent increase in *SEMA3A* expression (Fig. 2c,d). In contrast, human LSECs of the same donor treated with oleic acid showed neither a dose- nor a time-dependent elevation of *SEMA3A* expression (Fig. 2e,f), except for the 24 h time point. Of note, FFA treatment with either 0.5 mM palmitic or 0.5 mM oleic acid for 18 h reduced the viability of LSECs by less than 10%, as assessed by flow cytometry (Supplementary Information). Further, a more than twofold higher expression of *SEMA3A* after 24 h treatment with 0.5 mM palmitic acid could be observed in LSECs from a female human donor (QC-29B15F09) in two independent experiments (Extended Data Fig. 2a). Since high SEMA3A concentrations have been associated with altered F-actin stress fibers in endothelial cells in a previous study^29^, we also treated LSECs with 0.75 mM palmitic acid for 24 h, stained with phalloidin-FITC and found an increase of F-actin stress fibers in treated LSECs (Supplementary Information).

After identifying palmitic acid as a *SEMA3A*-stimulating factor, we next explored potential (lipid-regulated) transcription factors that drive *SEMA3A* expression in LSECs. We utilized the software CiiiDER^44^ and observed a total of 454 transcription factor binding sites between 1,500 bp upstream and 500 bp downstream of the *SEMA3A* transcription start site (Extended Data Fig. 2b and Supplementary Information). This region included the binding sites of several lipid-regulated transcription factors. Of note, a binding site for cAMP responsive element binding protein 1 (CREB1) was also predicted (Extended Data Fig. 2b). As palmitic acid has been shown to induce cAMP synthesis^45^, we treated human LSECs with 100 µM of the adenylyl cyclase agonist forskolin (FSK) to specifically elevate intracellular cAMP levels. We observed an eightfold increase in *SEMA3A* expression (Extended Data Fig. 2c,d), which could not be observed in the human hepatoma cell line HepG2. Hence, we conclude that palmitic acid promotes *SEMA3A* expression and alters the F-actin cytoskeleton in LSECs with a putative role of cAMP-dependent signaling.

### SEMA3A reduces fenestrae frequency and LSEC porosity

**Fig. 3:**
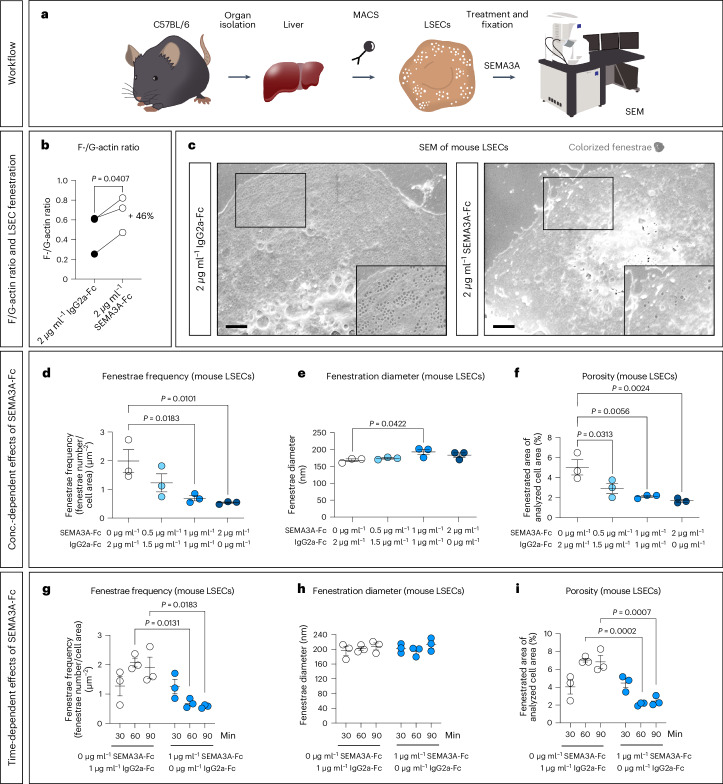
SEMA3A defenestrates LSECs in a concentration- and time-dependent manner. **a**, General workflow for LSEC experiments. **b**, F-actin/G-actin quantification in lysates from LSECs treated with IgG2a-Fc or SEMA3A-Fc (*n* = 3 independent LSEC isolations). **c**, Representative SEM images of LSECs treated for 1 h with SEMA3A-Fc and/or IgG2a-Fc. Brightness and contrast have been adjusted to enhance visibility. The fenestrae were colorized with a digital charcoal pencil for better visualization. Scale bars, 2 µm. **d**–**f**, Analysis of fenestrae frequency (**d**), diameter (**e**) and porosity (**f**) of LSECs treated for 1 h with SEMA3A-Fc and/or IgG2a-Fc concentrations as indicated (*n* = 3 independent experiments). The 1 µg ml^−1^ SEMA3A-Fc values are from the experiment shown below. **g**–**i**, Analysis of fenestrae frequency (**g**), diameter (**h**) and porosity (**i**) of LSECs treated with 1 µg ml^−1^ SEMA3A-Fc or IgG2a-Fc for 30, 60 or 90 min (*n* = 3 independent LSEC isolations). For statistical analysis a two-tailed paired Student’s *t*-test was performed in **b**, a one-way ANOVA with multiple comparisons (Dunnett’s post hoc test) in **d**–**f** and a two-way ANOVA with multiple comparisons (Tukey’s post hoc test) in **g**–**i**. For each condition, at least five images (taken from different LSECs) per experiment were analyzed. In all graphs data points and mean ± s.e.m. are presented. Source data

**Extended Data Fig. 3:**
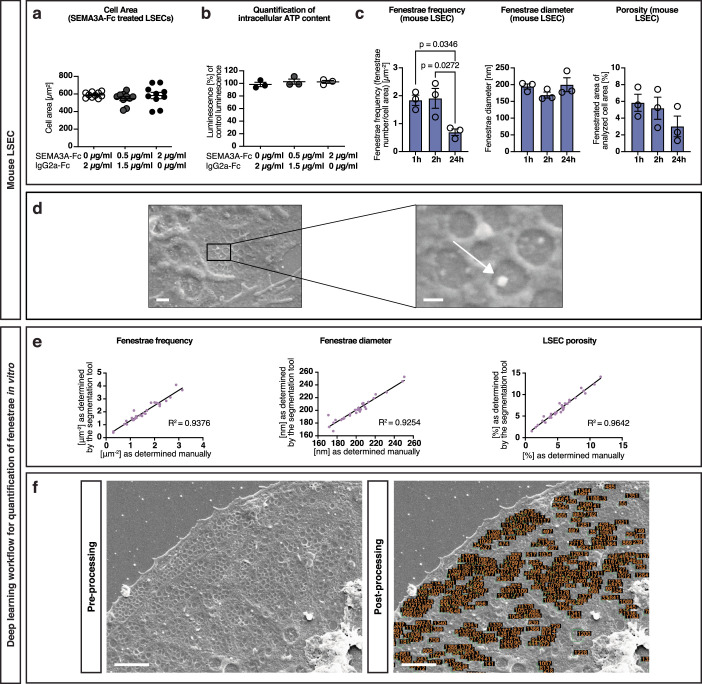
LSEC control experiments and deep learning workflow for quantification of LSEC porosity. **a**, Effect of different SEMA3A-Fc concentrations on LSEC size. Cells were cultured for 4 h, starved for 1 h, and treated with SEMA3A-Fc for 1 h. After fixation, phalloidin was used to stain F-actin fibers, and DAPI was used to stain cell nuclei. Cells were imaged using an Axioscope (Zeiss) and the NIS-Elements imaging software, and 10 images of each condition were obtained and analyzed using the Fiji image processing package. Per image, the cell size of at least 26 cells was measured. **b**, The CellTiter-Glo® Cell Viability Assay (Promega) was performed after SEMA3A-Fc treatment of isolated LSECs to determine the amount of ATP present (n = 3 independent LSEC isolations). **c**, Mouse LSEC were isolated and cultured in EBM-2 media for 1, 2 or 24 h, after 4 h pre-culture. Fenestrae were analyzed for their frequency and diameter; LSEC porosity was also determined. For each condition, 10 images (taken from different LSECs) were analyzed per experiment (n = 3 independent LSEC isolations). **d**, LSECs were isolated and incubated in EBM-2 media for 4 h. An arrow points to a potential magnetic bead located within a fenestra. Scale bars = 400 nm (left) and 100 nm (right, n = 1 LSEC isolation). **e**, Correlation analyses of 30 images that were analyzed either manually or using a deep learning workflow, for fenestrae frequency, fenestrae diameter and LSEC porosity. Each dot represents one image analyzed. **f**, Example image of LSEC pre- and post-processing (output file) as received by the deep learning workflow, scale bars = 2 µm. A one-way ANOVA with multiple comparisons was used for statistical analysis, and statistical significance was corrected for multiple comparisons using a Dunnett´s *post hoc test* in (**a**) and (**b**), and a one-way ANOVA with a Tukey´s *post hoc* test was used to test for statistical significance in (**c**). In graphs (**a**-**c**) individual data points and mean ± SEM are presented. Source data

Fenestrae are surrounded by tubulin and actin filaments^2,3^. As SEMA3A has been reported to regulate actin and tubulin dynamics^18,46^, and as palmitic acid alters the F-actin cytoskeleton in LSECs (Supplementary Information), we asked whether SEMA3A links hyperlipidemia to defenestration of hepatic sinusoids. Therefore, the effect of SEMA3A, applied as a recombinant mouse SEMA3A fused to a mouse IgG2a part (SEMA3A-Fc), was investigated in cell culture experiments with mouse LSECs. More specifically, alterations in the F-actin cytoskeleton and LSEC fenestration (diameter and frequency of fenestrae) were analyzed (Fig. 3a). First, we studied whether SEMA3A-Fc affected the ratio of F-actin to free globular-actin (G-actin) in magnetic-activated cell sorting (MACS)-isolated mouse LSECs after 1 h of treatment. Western blot analyses revealed a higher F-actin to G-actin ratio in SEMA3A-Fc-treated versus IgG2a-Fc-treated control LSECs (Fig. 3b), indicating that SEMA3A alters the F-actin cytoskeletal dynamics in LSECs. Next, we assessed the effects of SEMA3A on LSEC fenestration by treating MACS-isolated mouse LSECs with different concentrations of SEMA3A-Fc protein versus IgG2a-Fc control protein for 1 h (Fig. 3a,c and Extended Data Fig. 3a,b). This rather short duration was chosen to avoid a cell culture-dependent defenestration observed in LSECs around 24 h after MACS (Extended Data Fig. 3c). Before fenestrae quantification, we confirmed that residual magnetic beads did not influence identification of fenestrae, as they were different in size and appearance (Extended Data Fig. 3d). The frequency and diameter of fenestrae as well as LSEC porosity (∑ fenestrae area/analyzed cell area) were quantified in images taken from SEMA3A-Fc- and IgG2a-Fc control-treated LSECs by scanning electron microscopy (SEM) (Fig. 3d–f). For an unbiased image analysis, we developed a deep-learning workflow to quantify fenestrae number and diameter, which reduced the time required for quantification from days to minutes per experiment and showed strong correlations with manual analyses (average *R*^2^ = 0.9473; Extended Data Fig. 3e,f). Notably, treatment of mouse LSECs with SEMA3A-Fc resulted in a substantial dose-dependent reduction of fenestrae frequency of up to 73% (Fig. 3c,d). The average diameter of fenestrae remained unchanged after SEMA3A-Fc treatment, except for treatment with 1 µg ml^−1^ SEMA3A-Fc, which slightly increased the fenestrae diameter (Fig. 3e); however, the reduced fenestrae frequency (or defenestration) was enough to cause a dose-dependent loss of LSEC porosity of up to 66% (Fig. 3f). The observed SEMA3A-Fc-induced defenestration was not the result of cell contraction or energy depletion as neither cell area nor ATP concentrations of LSECs were altered upon treatment with SEMA3A-Fc when compared to the IgG2a-Fc control (Extended Data Fig. 3a,b).

We next treated LSECs with 1 µg ml^−1^ SEMA3A-Fc (or 1 µg ml^−1^ control IgG2a-Fc) for 30, 60 or 90 min and observed a time-dependent SEMA3A-Fc-mediated reduction of fenestrae frequency and LSEC porosity (Fig. 3g–i). Neither fenestrae frequency and diameter nor porosity changed after 30 min of SEMA3A-Fc treatment. In contrast, while fenestrae frequency and LSEC porosity increased after culturing LSECs for 60 min under IgG2a-Fc control culture conditions (a likely result of the recovery of LSECs from the MACS isolation process), a significant reduction of fenestrae frequency and porosity was visible after 60 and 90 min of SEMA3A-Fc treatment (Fig. 3g,i). In contrast to the fenestrae frequency, the fenestrae diameter remained unchanged during treatment with SEMA3A-Fc at all time points (Fig. 3h). Our experiments therefore suggest that SEMA3A reduces LSEC porosity by decreasing the frequency (rather than the diameter) of fenestrae.

### NRP1 is essential for SEMA3A-mediated defenestration of LSECs

**Fig. 4:**
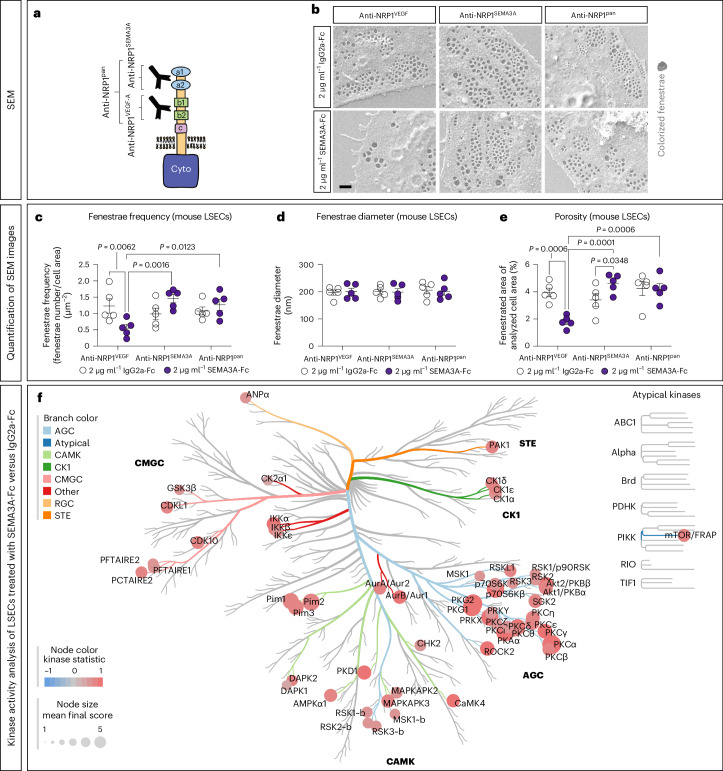
Blocking NRP1 reduces SEMA3A-induced LSEC defenestration that involves activation of multiple kinases. **a**, Schematic illustration of the NRP1 receptor and the binding sites of the anti-NRP1^VEGF^, anti-NRP1^SEMA3A^ or anti-NRP1^pan^ antibodies^47^. **b**, SEM images of LSECs first treated with anti-NRP1^VEGF^, anti-NRP1^SEMA3A^ or anti-NRP1^pan^ for 1 h and subsequently with either SEMA3A-Fc or IgG2a-Fc for 1 h. Brightness and contrast have been adjusted to enhance visibility. The fenestrae were colorized with a digital charcoal pencil for better visualization. Scale bar, 500 nm. **c**–**e**, Analysis of fenestrae frequency (**c**), diameter (**d**) and porosity (**e**) of LSECs that were first treated with either anti-NRP1^VEGF^, anti-NRP1^SEMA3A^ or anti-NRP1^pan^ for 1 h, and subsequently treated with either SEMA3A-Fc or IgG2a-Fc for 1 h. For statistical analysis a two-way ANOVA with multiple comparisons (Tukey’s post hoc test) was performed. For each condition, at least five images (taken from different LSECs) were analyzed per experiment (*n* = 5 independent LSEC isolations). In all graphs data points and mean ± s.e.m. are presented. **f**, Kinase activity profiling after UKA with a median final score of >1.2 taken as the threshold cutoff. For this assay, MACS-isolated mouse LSECs were treated with 1 µg ml^−1^ SEMA3A-Fc or IgG2a-Fc for 10 min. The data are visualized using a CORAL Kinome tree, where the color of a branch indicates the kinase family, the node color indicates the kinase statistic and the node size indicates the mean final score (mean specificity score + mean significance score). TK, tyrosine kinase group; CMGC, CDK, MAPK, GSK and CK2 kinase group; TKL, tyrosine kinase-like (TKL) group; STE, STE group kinases; CK1, casein kinase 1; AGC, protein kinase A, G and C group; CAMK, calcium/calmodulin-regulated kinase group; ABC1, ABC1 domain containing kinase; Alpha, alpha kinase group; Brd, bromodomain proteins; PDHK, pyruvate dehydrogenase kinase group; PIKK, phosphatidyl inositol 3′ kinase-related kinase group; RIO, RIO kinase group; TIF1, transcriptional intermediary factor 1. Source data

SEMA3A and VEGF-A can both bind to NRP1, but to different subdomains^47^. To find out whether NRP1 is required for the defenestrating effect of SEMA3A, we pretreated mouse LSECs with three different blocking antibodies against NRP1: (1) an antibody that blocks the VEGF-A binding domain of NRP1 (anti-NRP1^B^ or anti-NRP1^VEGF^)^47^; (2) an antibody blocking the SEMA3A-binding domain of NRP1 (anti-NRP1^A^ or anti-NRP1^SEMA3A^)^47^; and (3) a commercial antibody blocking the complete extracellular domain of NRP1 (anti-NRP1^pan^) (Fig. 4a). We found that SEMA3A-Fc led to a reduction of fenestrae frequency in the presence of anti-NRP1^VEGF^ antibodies (Fig. 4b,c). In contrast, SEMA3A-Fc-mediated defenestration was reduced in LSECs in the presence of either anti-NRP1^SEMA3A^ or anti-NRP1^pan^ (Fig. 4b–e). Again, fenestrae diameter was not affected by SEMA3A signaling (Fig. 4d) and consistent with the observed changes in fenestrae frequency (Fig. 4c), LSEC porosity was also dependent on the binding of SEMA3A-Fc to the SEMA3A-binding domain of NRP1 rather than its VEGF-A binding domain (Fig. 4e). We conclude that SEMA3A selectively induces LSEC defenestration by binding to NRP1.

### SEMA3A inhibits LSEC fenestration via LIMK1

To gain insights into the downstream signaling of SEMA3A, we measured kinase activities in primary mouse LSECs in an unbiased manner. Specifically, we treated mouse LSECs with IgG2a-Fc versus SEMA3A-Fc for 10 min and then performed a kinase activity profiling using the PamGene PamChip technology that measured the overall activity of 196 protein tyrosine kinases (PTKs) and 144 serine-threonine kinases (STKs). The profiling is based on measuring the phosphorylation of target peptides followed by an in silico upstream kinase analysis (UKA) to identify the kinases responsible for the phosphorylation. A total of 54 STKs belonging to different STK families were identified to be activated by SEMA3A (Fig. 4f and Supplementary Tables 2 and 3), whereas PTKs were largely unaffected. Notably, several of the identified SEMA3A-regulated STKs were reported to affect the F-actin cytoskeleton, such as PAK1 (p21 (RAC1) activated kinase 1 (ref. ^48^)) and ROCK2 (rho-associated, coiled-coil-containing protein kinase 2 (ref. ^49^); Fig. 4f).

**Fig. 5:**
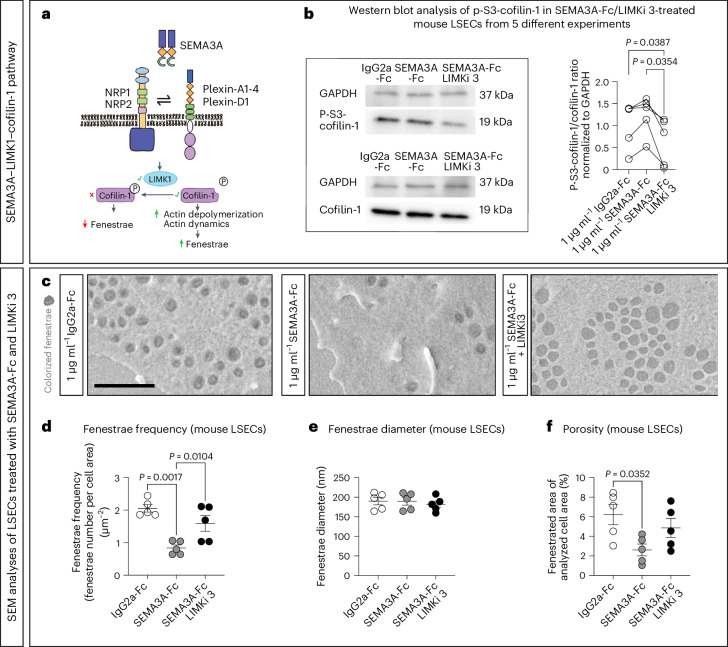
LIMK1 activity is required for SEMA3A-induced defenestration of mouse LSECs. **a**, Schematic illustration of SEMA3A signaling. Upon SEMA3A binding to NRP1, NRP1 forms a holoreceptor complex with a plexin, which acts as the signal-transducing unit. Through a signaling cascade, LIMK1 is activated and catalyzes the phosphorylation of cofilin-1. Cofilin-1 is an actin depolymerization factor, which is de-activated upon phosphorylation at its serine 3 (S3). Thus, less actin is depolymerized, resulting in a less dynamic actin network and, subsequently, fewer fenestrae. **b**, Western blots of mouse LSEC protein lysates (*n* = 5 independent LSEC isolations). LSECs were pretreated with either DMSO or LIMKi 3, a LIMK1 inhibitor, and then treated with either SEMA3A-Fc or IgG2a-Fc. For the analysis, cofilin-1 and p-S3-cofilin-1 were normalized to GAPDH and then put into relation of each other (p-S3-cofilin-1 to cofilin-1). **c**, Representative SEM images of mouse LSECs pretreated with either DMSO or LIMKi 3 and then treated with either SEMA3A-Fc or IgG2a-Fc. The fenestrae were colorized with a digital charcoal pencil for better visualization. Scale bar, 1 µm. Brightness and contrast have been adjusted to enhance visibility in **b**,**c**. **d**–**f**, Analyses of fenestrae frequency (**d**) and diameter (**e**) as well as porosity (**f**) of mouse LSECs pretreated with LIMKi 3 or DMSO and subsequently treated with SEMA3A-Fc or IgG2a-Fc, as indicated. For each condition, ten images (taken from different LSECs) were analyzed (*n* = 5 LSEC isolations). For statistical analysis, a one-way ANOVA with multiple comparisons (Tukey’s post hoc test) was performed in **b**,**d**–**f**. In all graphs, data points and mean ± s.e.m. are presented. Source data

LIM domain kinase 1 (LIMK1) is directly downstream of ROCK2 and PAK1 (refs. ^50,51^) and inactivates cofilin-1 by Ser3-phosphorylation (Fig. 5a), thereby reducing the actin-network dynamics (needed for maintaining fenestrae)^52^. To test whether SEMA3A regulates cofilin-1 in mouse LSECs, we treated the latter with SEMA3A-Fc or IgG2a-Fc for 1 h and quantified the Ser3-phosphorylated cofilin-1 (hereafter referred to as p-S3-cofilin-1) to total cofilin-1 ratio by western blot analyses. Treatment of LSECs with SEMA3A-Fc led to a higher ratio of p-S3-cofilin-1/total cofilin-1 compared to LSECs treated with an equal amount of IgG2a-Fc (Fig. 5b). To corroborate our findings, we repeated the treatment of LSECs with SEMA3A-Fc, but added LIMKi 3, a potent LIMK1 inhibitor^53^. In the presence of this inhibitor, the p-S3-cofilin-1/total cofilin-1 ratio was even lower compared to the untreated cells (Fig. 5b). Further, inhibition of LIMK1 was found to attenuate SEMA3A-induced reduction of fenestrae frequency and LSEC porosity but did not change the fenestrae diameter (Fig. 5c–f). We conclude that SEMA3A activates several STKs, including ROCK2 and PAK1, and requires LIMK1 to fully induce defenestration of LSECs (Fig. 5a).

### Heterozygous deletion of *Sema3a* increases fenestrae number

**Fig. 6:**
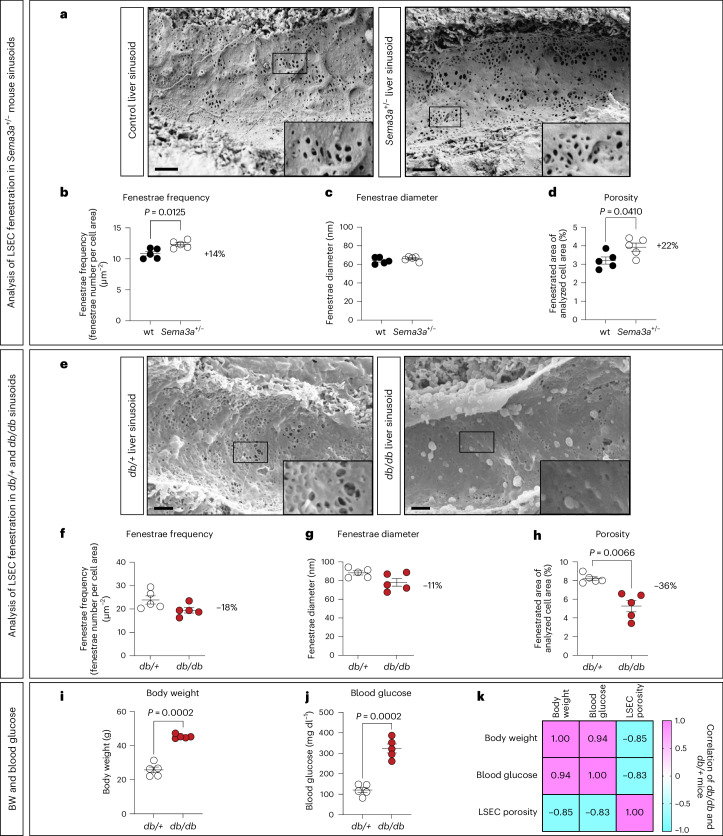
Opposing effects of *Sema3a* deletion and *Lepr* mutation on LSEC porosity. **a**, SEM images of liver sinusoids in 29-week-old male control and *Sema3a*^+/−^ mice kept on chow diet. Scale bars, 1 µm. **b**–**d**, Analysis of fenestrae frequency (**b**), diameter (**c**) and LSEC porosity (**d**) in liver sinusoids from *Sema3a*^+/−^ and control (wt) mice (*n* = 5 mice per genotype). **e**, SEM images of liver sinusoids in 10-week-old male *db*/+ and *db/db* mice. Scale bars, 1 µm. **f**–**h**, Analysis of fenestrae frequency (**f**), diameter (**g**) and LSEC porosity (**h**) in liver sinusoids of *db*/+ (control) and *db/db* mice (*n* = 5 mice per genotype). **i**,**j**, Body weight (**i**) and blood glucose concentration (**j**) of *db*/+ versus *db/db* mice (*n* = 5 mice each). **k**, Correlation matrix showing Pearson correlation coefficients for pairwise comparisons between the following variables: body weight, blood glucose and LSEC porosity in the combined cohort of *db*/+ and *db/db* mice. For statistical analysis in **b**–**j**, a two-tailed unequal variances *t*-test was performed. In all graphs individual data points and mean ± s.e.m. are presented. Source data

**Extended Data Fig. 4:**
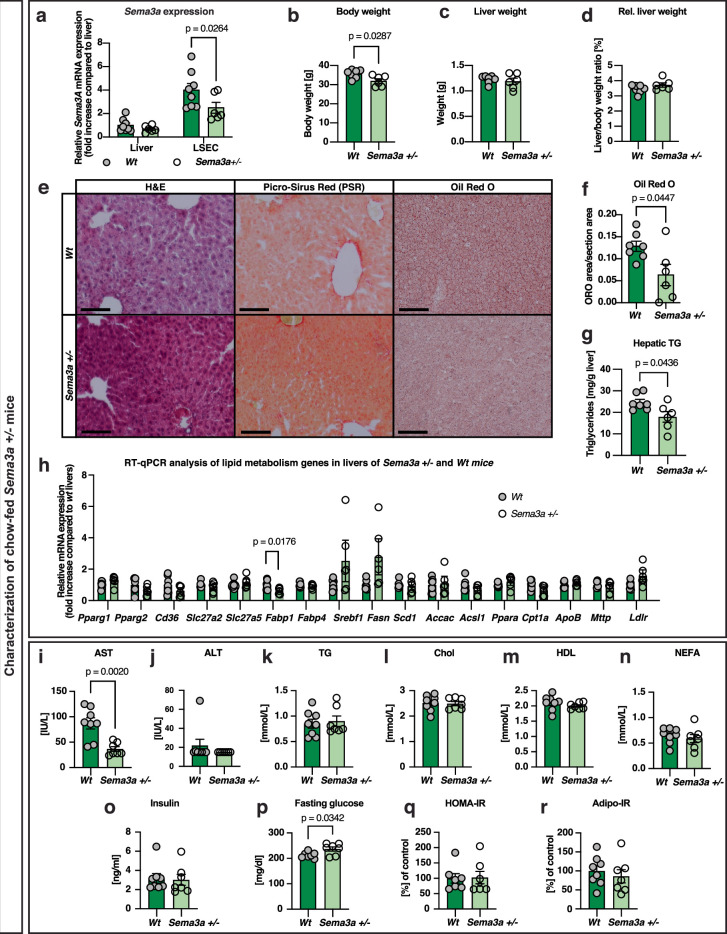
Lower hepatic fat content in chow-fed *Sema3a +/-* mice compared to controls. **a**, *Sema3a* expression in the liver and CD146-positive LSEC from male *Sema3a* +/- mice and controls at the age of 14 weeks (n = 6 *Sema3a* +/- and n = 8 *Wt* mice). **b**, Body weight (BW). **c**, Liver weight. **d**, Relative liver weight (% of BW). **e**, H&E, Picro-Sirius Red (PSR) and Oil Red O (ORO) staining; scale bars = 100 µm. **f**, Densitometric quantification of ORO staining on liver sections. **g**, Hepatic triglycerides (TG). **h**, RT-qPCR analysis of genes involved in lipid metabolism in liver tissue from fasted mice. Please note that male, chow-fed 35–38-week-old mice (fasted for 4 h) were used for (**b**-**h**, n = 7 *Wt* and n = 6 *Sema3a* +/- mice). **i-n**, Serum transaminase and lipid profile, that is AST, ALT, TG, total cholesterol (Chol), HDL, non-esterified fatty acids (NEFA) and **o**, Serum insulin. **p**, Fasting blood glucose concentration. **q**, HOMA-IR and **r**, Adipo-IR as measured in serum from 25–30−week-old mice (**i**-**o**,**r**, n = 8 *Wt*/n = 7 *Sema3a* +/-; **p**, **q**, n = 7 per genotype). AST/ALT values displayed as ‘under 15’ were defined as 15 U/L. A repeated measures two-way ANOVA with Sidak´s *post hoc* test was used to test for statistical significance in (**a**), and a two-tailed unequal variances *t-*test was performed in (**b**-**r**). In all graphs, individual data points and mean ± SEM are presented. Source data

**Extended Data Fig. 5:**
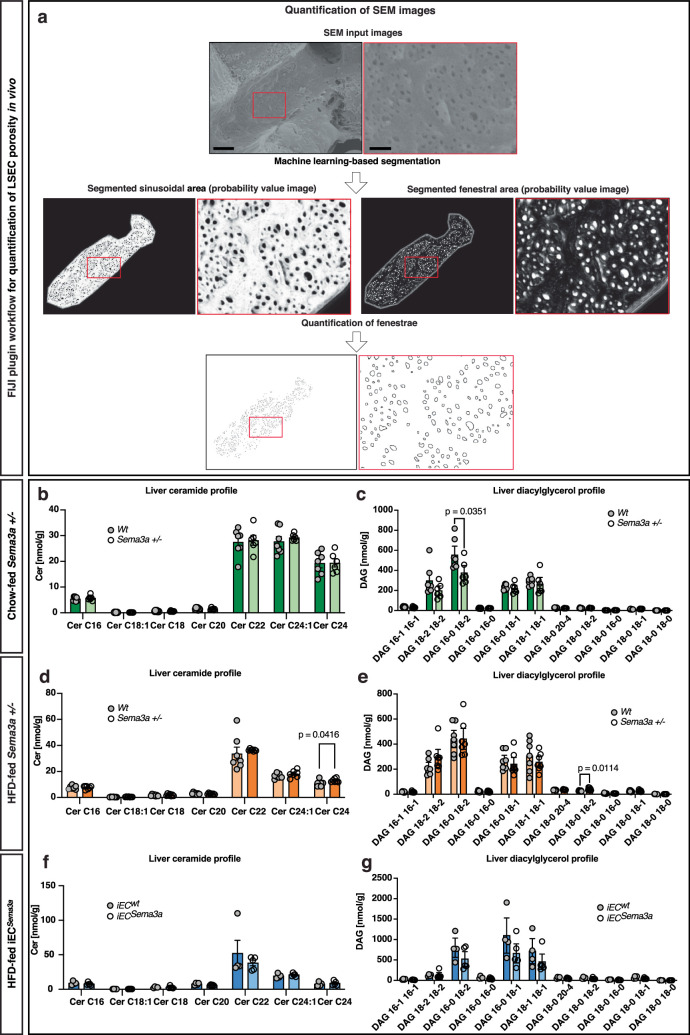
Analysis of in vivo LSEC porosity and targeted lipidomics. **a**, Representative images of SEM input images (upper panels), calculated probability maps using a machine-learning based approach (WEKA segmentation, middle panels), and outlines (lower panels) used to quantify fenestrae diameter as well as fenestrae frequency, scale bar = 2 µm for the input image (left); 500 nm for the magnification (right). **b**, Liver ceramide (Cer) and **c**, Liver diacylglycerol (DAG) profile of chow-fed 35–38-week-old male mice (n = 7 *Wt* and 6 *Sema3a +/-* mice). **d**, Liver Cer and **e**, Liver DAG profile of diet-induced obese (DIO) control and DIO *Sema3a +/-* mice kept on a HFD for 20 weeks (n = 7 mice of each genotype). **f**, Liver Cer and **g**, Liver DAG profile of DIO *iEC*^*Sema3a*^ and DIO *iEC*^*wt*^ mice kept on HFD for 20 weeks (with tamoxifen injections after 10 weeks of HFD; n = 4 *iEC*^*wt*^ and 5 *iEC*^*Sema3a*^ mice). For statistical analysis a two-tailed unequal variances *t-*test was performed. In all graphs, individual data points and mean ± SEM are presented. Source data

As our gain-of-function experiments revealed that SEMA3A lowers frequency of fenestrae in LSECs, we next asked whether, in turn, deletion of *Sema3a* increases the fenestrae frequency in LSECs. Due to high perinatal lethality of homozygous *Sema3a*^−/−^ (knockout) mice^54,55^, we analyzed adult heterozygous *Sema3a*^+/−^ mice and their wild-type littermates. *Sema3a*^+/−^ mice were viable, showed no obvious phenotypic differences from their control littermates, and displayed an approximate 40% reduction in LSEC *Sema3a* mRNA compared to wild-type controls (Extended Data Fig. 4a). To analyze the LSEC ultrastructure in these mice, we prepared liver samples from adult (29-week-old) *Sema3a*^+/−^ mice and littermate controls for SEM (Fig. 6a). Sinusoids of *Sema3a*^+/−^ mice were characterized by fenestrated LSECs and not easily distinguishable from control sinusoids at a cellular level. To quantify fenestrae frequency and diameter as well as porosity of hepatic sinusoids, we developed a fenestrae-detecting plugin for liver sinusoids based on a dataset-trained-classifier segmentation algorithm by using the image analysis software Fiji^56,57^. This plugin generated probability maps for surface area and fenestrae area (Extended Data Fig. 5a), which were used to quantify fenestrae diameter and frequency as well as LSEC porosity in liver tissue from *Sema3a*^+/−^ and wild-type littermate controls.

Notably, heterozygous deletion of *Sema3a* increased fenestrae frequency by 14% compared to wild-type littermates (Fig. 6b), whereas the fenestrae diameter was only slightly increased (Fig. 6c). This added up to an increase of 22% in LSEC porosity in *Sema3a*^+/*−*^ mice compared to their wild-type littermate controls (Fig. 6d). We then compared these results to LSEC fenestration of 10-week-old *db/db* and *db*/+ control mice (Fig. 6e), as LSECs from *db/db* mice at around this age (12 weeks) had a close to fourfold increase in *Sema3a* expression (Fig. 1d). Conversely to *Sema3a*^+/*−*^ mice, *db/db* mice displayed a numeric decrease in fenestrae frequency and diameter (Fig. 6f,g), leading to a 36% reduction of LSEC porosity in *db/db* versus *db*/+ liver tissue (Fig. 6h). In addition, LSEC porosity negatively correlated with body weight and blood glucose concentrations (Fig. 6i–k). We conclude that in obese, diabetic mice, LSEC porosity is reduced, but that deletion of just one *Sema3a* allele even in non-diabetic mice increases LSEC porosity in a haplo-insufficient manner.

### *Sema3a* is haplo-insufficient for liver fat content in chow-fed mice

Fenestration of LSECs promotes bidirectional exchange of carbohydrates, lipids and lipoproteins between the bloodstream and hepatocytes^2,11,30^. Thus, we analyzed liver samples from male 35–38-week-old *Sema3a*^+/*−*^ mice kept on chow diet and compared these samples to those from their chow-fed littermate controls (Extended Data Fig. 4). *Sema3a*^+/*−*^ mice weighed 10% less than wild-type littermates, but liver weight and liver-to-body weight ratio were unchanged (Extended Data Fig. 4b–d). Oil Red O (ORO) staining on cryosections of liver tissue revealed a 51% reduction in lipid droplet area of liver tissue from *Sema3a*^+/*−*^ mice compared to that of controls (Extended Data Fig. 4e,f). This finding was corroborated by biochemical measurement of liver TGs (Extended Data Fig. 4g), revealing a reduced liver fat content in *Sema3a*^+/*−*^ mice. At the same time, we found no obvious histological changes with respect to macrovesicular steatosis or fibrosis between the genotypes, as assessed by hematoxylin and eosin (H&E) and Picro-Sirius Red (PSR) staining, which is in line with the chow-feeding (Extended Data Fig. 4e). We next analyzed a set of genes involved in lipid metabolism by RT–qPCR (Extended Data Fig. 4h)^1^. While only one gene (*Fabp1*) was significantly regulated by *Sema3a* in the liver of chow-fed mice, the numeric reduction in the expression of *Pparg2* (peroxisome proliferator-activated receptor γ) and *Cd36* (fatty acid translocase/cluster of differentiation 36) was of particular interest, as these two genes were found to be more strongly reduced in *Sema3a*-deficient mice on an HFD (see sections below). We also assessed metabolic biomarkers in the serum of 26–30-week-old mice (Extended Data Fig. 4i–r), but did not observe major changes, except for a 61% decrease in the activity of the liver damage marker aspartate aminotransferase (AST) and a slight increase in fasting blood glucose concentration. We conclude that in chow-fed mice, *Sema3a* is haplo-insufficient for promoting lipid accumulation in the mouse liver, coinciding with its inhibitory effect on LSEC fenestration.

### *Sema3a* is haplo-insufficient for hepatic steatosis in DIO mice

**Extended Data Fig. 6:**
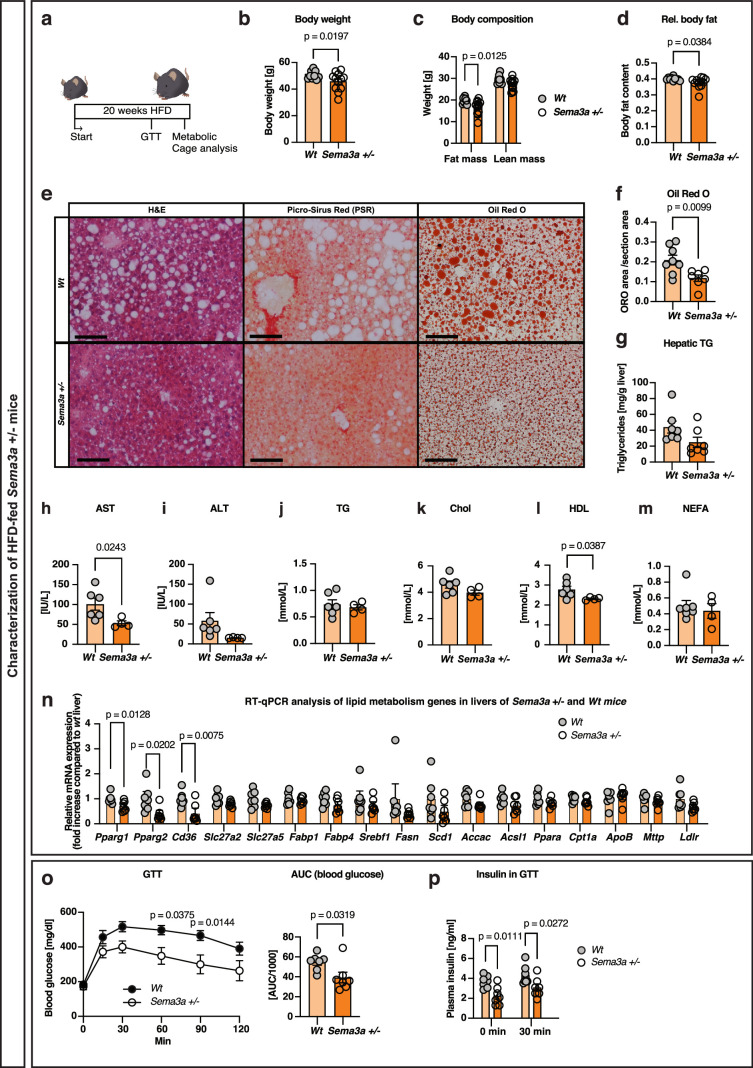
Lower hepatic fat content in diet-induced obese (DIO) *Sema3a +/-* mice. **a**, Experimental plot; **b**, Body weight; **c**, Body composition; **d**, Relative body fat (% of BW) in male DIO wild-type and DIO *Sema3a* +/- mice, both kept on a HFD for 20 weeks (**b**–**d**, n = 10 *Wt* and n = 12 *Sema3a* +/- male mice). **e**, H&E, Picro-Sirus Red (PSR) and Oil Red O (ORO) staining; scale bars = 100 µm. **f**, Densitometric quantification of hepatic ORO staining (n = 8 *Wt* and n = 7 *Sema3a* +/-). **g**, Liver triglycerides (TG, n = 7 male mice of each genotype). **h-m**, Serum transaminase and lipid profile, that is AST, ALT, TG, total cholesterol (Chol), HDL, non-esterified fatty acids (NEFA), in n = 6 (**h**, **i**, **j**, **k**, **l**) and n = 7 (**m**) *Wt* male DIO mice and n = 4 DIO *Sema3a* +/- mice kept on a HFD for 20 weeks. AST/ALT values displayed as ‘under 15’ were defined as 15 U/L. **n**, RT-qPCR analysis of genes involved in lipid metabolism in liver tissue from fasted mice. Please note that male, chow-fed 35–38-week-old mice (fasted for 4 h) were used (n = 7 per genotype). **o**, Intraperitoneal glucose tolerance test (GTT) and area under the curve (AUC), n = 7 mice of each genotype; **p**, Plasma insulin concentrations during the GTT, n = 7 mice of each genotype. For statistical analysis, a two-tailed, unequal variances *t-*test was performed in **b**, **d**-**n**, and **o** (for the AUC blood glucose). A repeated measures two-way ANOVA with Sidak´s *post hoc* test was used to test for statistical significance in **c**, **o** (for the GTT) and **p**. In all graphs, individual data points, mean ± SEM are presented. Source data

**Extended Data Fig. 7:**
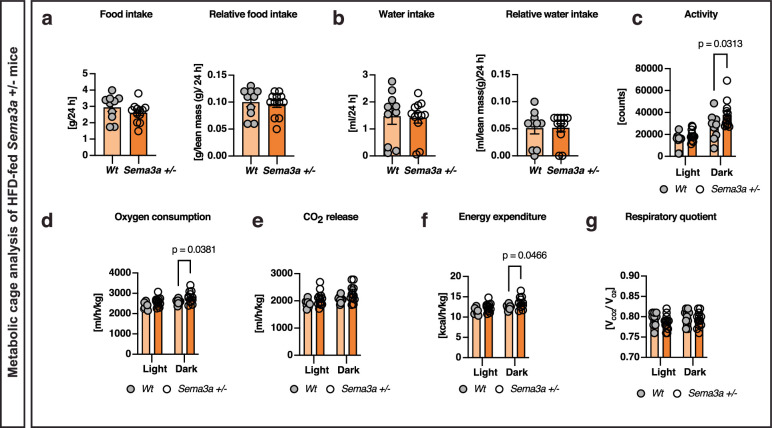
Metabolic cage analysis of diet-induced obese (DIO) *Sema3a +/-* mice. **a**, Absolute and relative food intake in 24 h, normalized to lean mass of DIO control mice and DIO *Sema3a* +/- mice. **b**, Absolute and relative water intake in 24 h, normalized to lean mass. **c**, Physical activity of mice quantified by summarizing photo sensor counts in x, y and z directions separately for day (Light) and night (Dark). **d**, Oxygen consumption (ml/h/kg body weight) and **e**, Carbon dioxide (CO_2_) release (ml/h/kg body weight); **f**, Energy expenditure (kcal/h/kg body weight). **g**, Respiratory quotient (V_CO2_/V_O2_). N = 10 *wt* and n = 12 *Sema3a* +/- mice (20 weeks kept on a high-fat diet) in (**a**-**g**). A two-tailed unequal variances *t*-test was used to assess statistical significance in (**a**, **b**); and a repeated measures two-way ANOVA with a Sidak´s *post hoc* test was used in (**c**-**g**). In all graphs, individual data points, mean ± SEM are presented. Source data

We next studied the degree of hepatic steatosis in liver tissue from DIO *Sema3a*^+/*−*^ and control mice after feeding them an HFD for 20 weeks (Extended Data Fig. 6a). Compared to control DIO mice, *Sema3a*^+/*−*^ DIO mice displayed a slightly lower body weight, lean and fat mass and relative body fat content as assessed by NMR (Extended Data Fig. 6b–d). In agreement with the reduced lipid content in livers of chow-fed mice, ORO staining of liver cryosections and biochemical TG measurement revealed a 44% reduction of hepatic fat content in liver tissue from *Sema3a*^+/*−*^ DIO mice compared to that of DIO controls (Extended Data Fig. 6e–g). While fibrosis was not detectable in either genotype, and most biomarkers were largely unchanged, AST plasma concentrations were lower in the blood taken from DIO *Sema3a*^+/*−*^ mice versus DIO control mice (Extended Data Fig. 6h–m). Metabolic cage analyses revealed a higher degree of physical activity and oxygen consumption compared to control mice, suggesting that in DIO *Sema3a*^+/*−*^ mice, peripheral tissues metabolize lipids that otherwise accumulate in the liver (Extended Data Fig. 7). The lower hepatic fat content in DIO *Sema3a*^+/*−*^ mice coincided with a reduced expression of *Pparg1* and *Pparg2* along with a reduced expression of their downstream target *Cd36* (Extended Data Fig. 6n).

We also quantified multiple ceramides and diacylglycerols (DAGs) in liver tissue from chow-fed and DIO mice, but without observing substantial differences, except that abundance of the very-long-chain ceramide Cer 24:0 (previously suggested to protect from liver steatosis^58^) was slightly higher in DIO *Sema3a*^+/*−*^ mice compared to their DIO controls and that the concentrations of several DAGs were slightly lower in the other two mouse models carrying a *Sema3a* deletion (Extended Data Fig. 5b–g). A glucose tolerance test (GTT) revealed a markedly improved glucose tolerance in DIO *Sema3a*^+/*−*^ mice compared to control DIO mice (Extended Data Fig. 6o). In addition, plasma insulin concentrations were significantly lower during the GTT, suggesting that the improved glucose tolerance in *Sema3a*^+/*−*^ mice was caused by a higher insulin sensitivity rather than an improved pancreatic islet function (Extended Data Fig. 6p). We conclude that in obesity, *Sema3a* is haplo-insufficient for promoting early stage MASLD.

### EC-specific deletion of *Sema3a* lowers hepatic steatosis

**Fig. 7:**
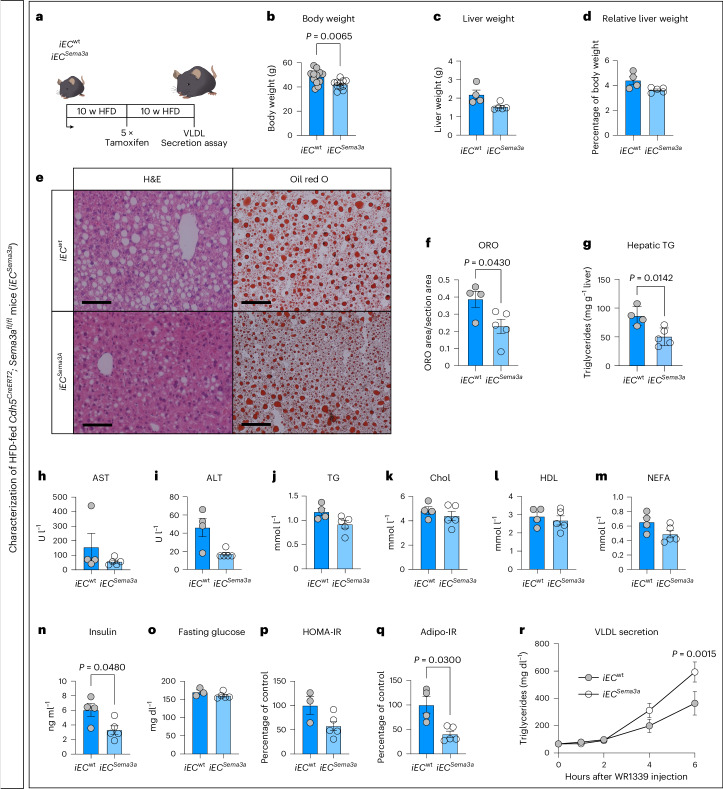
Lower hepatic fat content in DIO *iEC*^*Sema3a*^ mice compared to DIO *iEC*^wt^ mice. Analysis of *Cdh5-Cre*^ERT2^ × *Sema3a*^fl/fl^ (*iEC*^Sema3a^) and *Cdh5-Cre*^ERT2^ (*iEC*^wt^) mice kept on HFD for 20 weeks (with tamoxifen injections on 5 consecutive days after 10 weeks of HFD). **a**, Experimental plot. **b**, Body weight (BW). **c**, Liver weight. **d**, Relative liver weight (% of BW). **e**, H&E and ORO staining of liver sections. Scale bars, 100 µm. **f**, Densitometric quantification of liver ORO staining. **g**, Hepatic TGs. **h**–**m**, Transaminase and serum lipid profile (AST (**h**), ALT (**i**), TG (**j**), total cholesterol (Chol; **k**), high-density lipoprotein (HDL; **l**) and FFA/NEFA (**m**)). AST/ALT values displayed as ‘under 15 U l^−1^’ were defined as 15 U l^−1^. **n**, Serum insulin. **o**, Fasting blood glucose. **p**, HOMA-IR. **q**, Adipo-IR. *n* = 12 *iEC*^wt^ and *n* = 11 *iEC*^*Sema3a*^ mice (**b**); *n* = 4 *iEC*^wt^ and *n* = 5 *iEC*^*Sema3a*^ mice (**c**–**n**,**q**); *n* = 3 *iEC*^wt^ and *n* = 5 *iEC*^*Sema3a*^ mice (**o**,**p**) analyzed after 20 weeks of HFD (10 weeks after *Sema3a* deletion by tamoxifen). **r**, Measurement of VLDL (TG) secretion after injection of WR1339 (*n* = 12 *iEC*^wt^ and *n* = 11 *iEC*^*Sema3a*^ mice per genotype) after 18 weeks of HFD (around 8 weeks after *Sema3a* deletion by tamoxifen). For statistical analysis, two-tailed unequal variances *t*-tests were performed in **b**–**q**. A repeated measures two-way ANOVA with a Sidak’s post hoc test was used to test for statistical significance in **r**. In all graphs, individual data points and mean ± s.e.m. are presented. Source data

**Extended Data Fig. 8:**
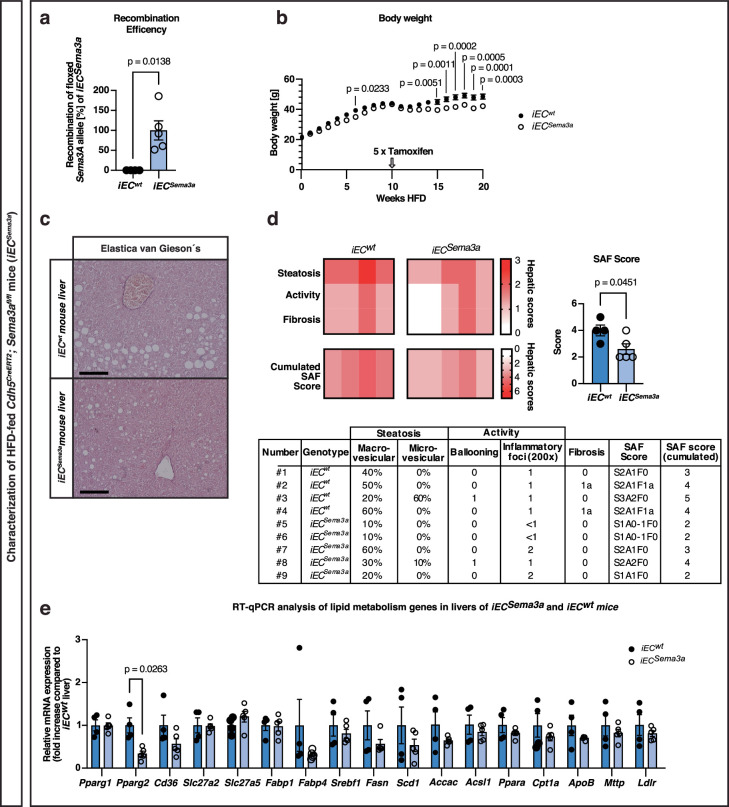
Lower SAF score in adult DIO mice after deletion of endothelial *Sema3a.* Analysis of diet-induced obese (DIO) *iEC*^*wt*^ mice and DIO *iEC*^*Sema3a*^ kept on HFD for 20 weeks (with tamoxifen injections after 10 weeks of HFD). **a**, Quantification of *Sema3a* allele recombination in liver tissue from DIO *iEC*^*wt*^ and DIO *iEC*^*Sema3a*^ mice as assessed by PCR. **b**, Body weight during exposure to HFD. **c**, Elastica van Gieson´s staining of paraffin sections of liver tissue to assess fibrosis; scale bars = 100 µm. **d**, Assessment of the grade of MASLD according to the steatosis, activity, fibrosis (SAF) score. **e**, RT-qPCR analysis of genes involved in hepatic liver metabolism. N = 4 *iEC*^*wt*^ and n = 5 *iEC*^*Sema3a*^ mice were analyzed for each genotype in (**a**, **c**-**e**) and n = 12 *iEC*^*wt*^ and n = 11 *iEC*^*Sema3a*^ mice in (**b**). For statistical analyses, two-tailed unequal variances *t*-tests were performed in (**a**, **d**, **e**), while a repeated measures two-way ANOVA with a Sidak´s *post hoc* test was used to test for statistical significance in (**b**). In all graphs, individual data points and mean ± SEM are presented. Source data

We next assessed whether reduction of SEMA3A signaling in mice with manifested hepatic steatosis could reduce hepatic fat content. Therefore, we generated *Cdh5-Cre*^ERT2^ *×* *Sema3a*^fl/fl^ mice (hence abbreviated as *iEC*^*Sema3a*^), as the *Cdh5-Cre*^*ERT2*^ strain is considered endothelial cell (EC)-specific and allows ablation of *Sema3a* in ECs of adult mice by tamoxifen injections^59^. More specifically, *iEC*^*Sema3a*^ mice and *Cdh5-Cre*^*ERT2*^ controls (abbreviated as *iEC*^wt^) were fed an HFD for 10 weeks to induce hepatic steatosis, followed by injections with tamoxifen to efficiently delete the *Sema3a* allele (Fig. 7a and Extended Data Fig. 8a). After recombination, mice were kept on HFD for another 10 weeks to finally investigate the effects of EC-specific deletion of *Sema3a* in the context of DIO. The *iEC*^*Sema3a*^ mice weighed 13% less than *iEC*^wt^ mice and liver weight and liver-to-body weight ratio were only slightly reduced (Fig. 7b–d and Extended Data Fig. 8b). In agreement with the results from global *Sema3a*^+/*−*^ mice on chow diet and HFD (Extended Data Figs. 4e–g and 6e–g), hepatic fat content in *iEC*^*Sema3a*^ mice was reduced compared to *iEC*^wt^ mice after 20 weeks of HFD feeding (Fig. 7e–g). Moreover, histological MASLD grading of liver sections was performed as described^60^. It revealed a decreased steatosis, activity and fibrosis (SAF) score for *iEC*^*Sema3a*^ liver tissue (Extended Data Fig. 8c,d), which was mainly due to reduced macrovesicular steatosis. The latter was evident on histological staining of liver sections (Fig. 7e and Extended Data Fig. 8c). Analysis of liver transaminases and lipids further revealed numerically reduced serum concentrations of the liver damage marker alanine aminotransferase (ALT) in *iEC*^*Sema3a*^ mice (Fig. 7h–m). Similar to the situation found in DIO *Sema3a*^+/*−*^ mice (Extended Data Fig. 6n), RT–qPCR also revealed a downregulation of *Pparg2* in the liver of DIO *iEC*^*Sema3a*^ mice compared to that from *iEC*^wt^ control mice (Extended Data Fig. 8e), whereas liver ceramide and DAG species were largely unchanged, except for slightly lower DAG levels, consistent with the notion that *Sema3a* mainly affects early development of MASLD (Extended Data Fig. 5f,g). Further, a numeric reduction in HOMA-IR and reduction in Adipo-IR along with reduced insulin concentrations at normal blood glucose concentrations indicated that whole-body and adipose tissue insulin sensitivity was improved in *iEC*^*Sema3a*^ versus *iEC*^wt^ mice (Fig. 7n–q).

As we hypothesized that a higher LSEC porosity facilitates lipid export from liver tissue into the bloodstream, we next quantified VLDL secretion in *iEC*^*Sema3a*^mice and *iEC*^wt^ control mice, both kept on HFD for 18 weeks (8 weeks after *Sema3a* gene recombination; Fig. 7r). As previously described^22^, we injected the lipoprotein lipase (LPL) inhibitor Triton WR1339 and measured TG (as a proxy for VLDL) in the blood from fasted mice. Consistent with the notion of a higher VLDL secretion under conditions of higher LSEC porosity, DIO mice with an EC-specific deletion of *Sema3a* were found to secrete a larger amount of VLDL into the blood circulation than the tamoxifen-injected DIO control mice (Fig. 7r). These experiments show that deletion of endothelial *Sema3a* in adult DIO mice reduces early hepatic steatosis and improves VLDL secretion from the liver.

## Discussion

**Fig. 8:**
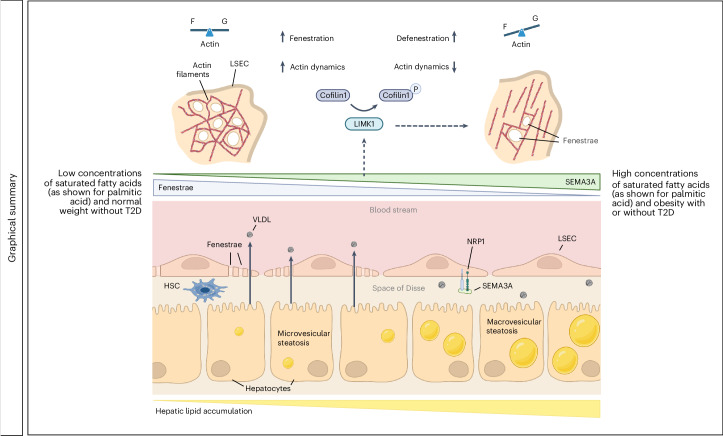
Model. Left side: in the setting of low physiological SEMA3A levels (as is the case at low concentrations of saturated fatty acids and normal BW without T2D), active cofilin-1 and normal F-actin cytoskeleton dynamics contribute to maintain a high frequency of fenestrae in LSECs. LSEC porosity facilitates bidirectional exchange of lipids between bloodstream and hepatocytes, such as the release of VLDL particles from hepatocytes into the blood circulation. Right side: in the setting of high SEMA3A levels (as is the case at high concentrations of FFAs and in DIO with or without T2D), the angiocrine signal SEMA3A acts via NRP1 on LSECs to activate multiple STKs, including LIMK1, which phosphorylates cofilin-1 to reduce F-actin cytoskeleton dynamics and fenestrae frequency as well as LSEC porosity. The reduced LSEC porosity lowers VLDL export from the hepatocytes into the blood and might contribute to lipid retention and macrovesicular steatosis in the hepatocytes. The resulting hepatic steatosis is an early event in MASLD that can subsequently (in concert with hepatic stellate cells; HSCs) progress to severe hepatic and cardiometabolic diseases. The figure was created with BioRender.com. Source data

In this study, we investigate the role of SEMA3A in LSEC defenestration that is associated with early development of MASLD, a disease with an estimated prevalence of at least 30% worldwide^61^. With respect to its pathogenesis, we show that *SEMA3A* is upregulated under conditions of high palmitic acid concentrations in female and male human LSECs and in multiple mouse models for MASLD. We further demonstrate that SEMA3A-Fc proteins result in robust defenestration of LSECs. In turn, using a number of different knockout mice for *Sema3a*, we show that a reduced expression of *Sema3a* results in more fenestrae and higher LSEC porosity. We conclude that SEMA3A contributes to defenestration of LSECs (Fig. 8). Along with the higher degree of LSEC fenestration and porosity, a lower degree of hepatic steatosis is observed. As we did not delete *Sema3a* selectively in LSECs (because no LSEC-specific *Cre* mouse line was used), we also consider the possibility that other types of ECs contribute to the positive outcome of the EC-specific deletion of *Sema3a*; however, hydrodynamic injections performed by Zhou et al. to selectively silence or overexpress *Nrp1* (coding for the co-receptor for SEMA3A) in the liver of DIO mice recently revealed that less NRP1 leads to reduced hepatic steatosis, whereas more NRP1 leads to increased hepatic steatosis^62^. These results are consistent with our proposal that silencing *Sema3a* affects the liver directly rather than exclusively via peripheral tissues such as adipose tissue (Fig. 8). As hepatocytes virtually lack the obligatory SEMA3A co-receptors NRP1 and NRP2, whereas LSECs express these proteins, as demonstrated by our current work and as previously reported^63^, SEMA3A likely acts in an autocrine manner on LSECs, reducing their porosity and inhibiting VLDL secretion from the liver to peripheral organs.

As SEMA3A-mediated microvascular alterations are likely to take place in peripheral and endocrine tissues as well (that were not the subject of this study), the metabolic phenotype observed must be considered in a larger context. For example, it is likely that the decelerated weight gain and increased energy expenditure after *Sema3a* deletion are triggered by alterations in endocrine and peripheral tissues, such as the adipose tissue^64^. Of note, all endocrine organs harbor a fenestrated microvasculature and could therefore be targeted by EC-derived SEMA3A^65^. Several neural cell types also express NRP1 as a co-receptor for SEMA3A^66^ and paracrine effects of SEMA3A on these cell types might further contribute to the observed metabolic phenotype. Still, liver-specific alterations alone can be sufficient for body weight loss^67,68^ and an improved liver sinusoidal fenestration facilitates delivery of VLDL from hepatocytes to peripheral tissues, as shown in this report, but might also affect the release of hepatokines from the liver^69^. In other words, while our report provides definitive evidence that SEMA3A in the microvascular endothelium promotes the early development of MASLD, understanding how SEMA3A affects systemic metabolism requires comprehensive investigations of other organs and cell types.

VEGF-A and SEMA3A are competitors, as they both share NRP1 as their co-receptor on ECs and recruit NRP1 to induce signaling via VEGFR2 and plexins (for example, plexin-A1)^47,70–72^, respectively. Disruption of VEGF-A signaling in mice was found to reduce fenestration and lipoprotein uptake^23^, whereas controlled overexpression of this growth factor was reported to reduce hepatic steatosis and extend the life-span of mice^73^. Based on these and other reports on the role of VEGF-A in LSEC fenestration and MASLD, it is likely that the ratio of VEGF-A and SEMA3A rather than one factor alone controls whether a liver remains fenestrated, attenuating hepatic steatosis or, alternatively, defenestrates, thus promoting early development of MASLD; however, from a pharmacologic point of view, blocking SEMA3A signaling may be more straightforward than activating a positive regulator such as VEGF-A and structural proteins that maintain fenestration^23,74^.

Both, in animal models for MASLD (with or without diabetes) and in primary human LSECs (from both male and female donors) treated with palmitic acid, expression of *Sema3a*/*SEMA3A* is substantially enhanced, revealing how this defenestrating (angiocrine) signal is induced by DIO. SEMA3A activates multiple different kinases, including PAK1 and ROCK2 that influence the F-actin cytoskeleton, in part via the LIMK1–cofilin-1 axis. Experiments with a LIMK1 inhibitor suggest that this kinase is required for a large part of the defenestration effect of SEMA3A. Notably, fenestrae are (at least in vitro) dynamic rather than static structures that require continuous F-actin remodeling^8,52^, which seems to be regulated by SEMA3A.

In sum, this report reveals a molecular mechanism by which DIO and saturated fatty acids trigger the defenestration of LSECs, an event observed at an early stage of MASLD^5^. The latter disease has a high prevalence and risk for progressing to MASH and serious complications such as fibrosis and cirrhosis and it also promotes cardiovascular diseases^75^. Our study therefore warrants further research on the SEMA3A–NRP1 signaling pathway and its potential targets to attenuate early MASLD development as an entry point for progression to life-threatening hepatic and cardiometabolic sequelae.

## Methods

### Experimental models and human donor information

Hepatocytes from various human donors were acquired from Thermo Fisher Scientific (HU4248, HU8296) and KaLy-Cell (S1426T). The donors were: female, 12 years old, white, body mass index (BMI) of 20.2, cause of death (COD) intracerebral hemorrhage-stroke (lot no. HU4248); male, 23 years old, white, BMI of 24.6, COD head trauma (lot no. HU8296); and female, 34 years old, white, BMI of 27.6, COD cholangiocarcinoma (lot no. S1426T). cDNA from these hepatocytes was obtained from elsewhere^32^. Human LSECs from different donors were purchased from PELOBiotech (PB-CH-153-5511). The donors were: female, 59 years old, white, BMI of 18, COD anoxia (QC-29B15F09) and male, 52 years old, white, BMI of 30.6, COD anoxia (QC-12B15F11).

Male C57BL/6J mice (Janvier), male C57BL/6N and male *db/db.BKS* (*BKS.Cg-Dock7*^*m*^ *+/+ Lepr*^*db*^*J*, JAX 000642), *ob/ob*.B6 (*B6.Cg-Lep*^*ob*^*/J*; Jackson Laboratories, JAX 000632) and control mice were used for LSEC isolations and gene expression studies. Male heterozygous *Sema3a* knockout mice (C57BL/6N background^54^) and male wild-type littermate control mice were used to study sinusoidal porosity, hepatic lipid content and metabolic parameters and were either fed with standard chow (Sniff, V1184-300; crude protein (N × 6.25) 23%; crude fat 6.1%; crude fiber 3.3%; crude ash 6.5%; starch 34.1%; sugar 5.1%; N free extracts 49.8%; energy from fat 16 kJ%; protein 27 kJ% and carbohydrates 57 kJ%) or HFD (D12492, Research Diets, energy from fat 60 kcal%; formulation: protein (200 g casein, Latic 30 Mesh and 3 g cysteine L), carbohydrates (125 g Lodex 10 and 72.8 g sucrose); fiber (50 g Solka Floc, FCC200); fat (245 g lard and 25 g soybean oil, USP), mineral (50 g S10026B); vitamin (2 g choline bitartrate and 1 g V10001C) and dye (0.05 g blue FD&C, Alum. Lake 35–42%)) and had free access to water.

For conditional vascular EC-specific deletion of *Sema3a*, *Cdh5-Cre*^*ERT2*^ *mice*^59^ were mated with *Sema3a*^fl/fl^ (backcrossed to C57BL/6J) mice^54^, fed with HFD (D12492, Research Diets) for 10 weeks, injected with 75 mg kg^−1^ body weight of tamoxifen (Sigma, T5648) in peanut oil (Sigma, P2144) for 5 consecutive days and fed with HFD for an additional 10 weeks. *Cdh5-Cre*^*ERT2*^ mice were used as controls and were treated equally. One mouse that experienced weight loss during the final days of the experiment was excluded from subsequent analysis. For recombination analysis, DNA from liver was extracted and a genotyping PCR was performed and analyzed by agarose gel electrophoresis. The band representing the recombined *Sema3a* allele (delta band) was quantified by densitometric analysis using Fiji^56^. Genotyping was performed according to previous studies^54,59^. All mice were held at 22 °C (±2 °C), 55% (±5%) humidity, lighting (6:00 to 18:00). The Animal Ethics Committee of the Landesamt für Natur, Umwelt und Verbraucherschutz Nordrhein-Westfalen (LANUV North Rhine-Westphalia, Germany, nos. 8.87-50.10.37.09.102; 81-02.04.2022.A187, 84.02.04.2017.A305 and 81-02.04.2019.A321) and the German Diabetes Center (DDZ) Institutional Animal Welfare Committee approved all animal experiments, which were conducted in accordance with German Animal Protection Laws.

### RNA isolation and RT–qPCR

To quantify gene expression in tissues or cells, mRNA was isolated using the RNeasy kit (QIAGEN). cDNA was synthesized using Oligo (dT) primers (Eurogentec) and MMLV reverse transcriptase (Promega) according to the suppliers’ instructions. qPCR was performed on a Mx3000P (Agilent Technologies) or Quantstudio 5 (Applied Biosystems) qPCR Machine using Brilliant III Sybr green (Agilent Technologies). To exclude the involvement of unspecific PCR products, –RT controls were performed and PCR melting curves of each PCR product were evaluated. Samples with faulty dissociation curves (more than two peaks) were excluded from further analysis. PCRs for all samples were run in triplicate. Relative gene expression was calculated according to Schmittgen and Livak^76^ using the formula 2^-(C(T) gene of interest- C(T) reference gene)^. Finally, individual samples were plotted as fold expression with respect to the mean of the control group. For additional visualization (Fig. 1i), PCR products were separated by gel electrophoresis analysis (2% agarose gel) and documented on a ChemiDoc XRS imaging system (Bio-Rad).

### Targeted lipidomics and triglyceride measurements

Diacylglycerols and ceramides were extracted from the liver and analyzed according to previous work^77^. Approximately 20 mg mouse liver were homogenized in 500 µl buffer cocktail (20 mM Tris-HCL, pH 7.4, 1 mM EDTA, 0.25 mM EGTA pH 7.0, 250 mM sucrose and protease and phosphatase inhibitor) using a tight-fitting glass Douncer (Wheaton). Internal standards were added to all samples. The resulting lipid phase was dried under a gentle flow of nitrogen and resuspended in methanol. For diacylglycerol and ceramide analysis, solid-phase extraction (Sep Pak Diol Cartridges; Waters) was performed. The resulting lipid phase was dried under a gentle flow of nitrogen and resuspended in methanol. The chromatographic separation of analytes was conducted using an Infinity 1290 Ultra-High Performance Liquid chromatography system (Agilent Technologies) and a reverse-phase Luna Omega C18 column, 50 × 2.1 mm, 1.6 µm (Phenomenex) operated at 50 °C. The injection volume was 1 µl. The analytes were measured as ammonium adducts (DAGs) or protonated adducts (CERs) using electrospray ionization and detected by multiple reaction monitoring on a triple quadrupole mass spectrometer (Agilent 6495; Agilent Technologies) operated in positive ion mode. Data analysis was performed using MassHunter Workstation software (Agilent Technologies) and Microsoft Excel.

Hepatic TGs were measured using a luciferase-based assay (Triglyceride-Glo Assay, Promega). In brief, approximately 25 mg mouse liver were homogenized in PBS and centrifuged. The supernatant was diluted (1:5 or 1:4) in PBS and measured as described in the manual provided.

### Treatment of human LSECs and HepG2 cells

LSECs were cultured in T75 flasks coated with Speed Coating Solution (PELOBiotech, PB-LU-000-0002-00) in microvascular EC growth medium supplemented with a microvascular EC growth kit enhanced (PELOBiotech, PB-MH-100-4099). For fatty acid treatments, LSECs (passage 4–6) were passaged in 12-well dishes coated with Speed Coating Solution (PELOBiotech, PB-LU-000-0002-00) and left to attach overnight. Thereafter, different concentrations of palmitic acid (Sigma-Aldrich, P5585), sodium oleate (Sigma, O7501) diluted with fatty acid-free BSA (Sigma-Aldrich, A7039, lot SLCB3395) or fatty acid-free BSA as control were added to the cells and incubated for 2, 6, 18 and 24 h. Finally, the medium was removed and the cells were collected in 350 µl RTL lysis buffer (QIAGEN) to isolate RNA.

For treatment with FSK LSECs (passage 4–7) were seeded into coated six-well plates at 500,000 cells per well, and on the following day incubated with 100 µM FSK or dimethylsulfoxide (DMSO) for up to 6 h. The cells were collected in 350 µl RLT lysis buffer (QIAGEN) to isolate RNA and perform RT–qPCR analysis. HepG2 cells (ATCC, HB-8065) were cultured in DMEM (1×) + GlutaMax (Gibco, cat. no. 21885-025) at 37 °C with 5% CO_2_ and treated in the same way as LSECs.

### Flow cytometry of human LSECs

Flow cytometry was performed to determine the frequency of dead LSECs after treatment with palmitic acid or BSA. After treatment of the cells, the medium was collected and adherent cells were detached by trypsinization and transferred into FACS tubes (Falcon, 352052). FACS tubes were centrifuged (400*g*, 5 min) and cells were washed with PBS (Gibco, 10010-015). The centrifugation step was repeated and FVS660 (BD Biosciences, 564405, 1:1,000 dilution) diluted in PBS was added for 15 min at room temperature in the dark. Cells were washed with PBS and centrifuged for 3 min at 400*g* twice. The cell pellet was resuspended in PBS and FVS660^+^ (dead) and FVS660^−^ (living) cells were determined using CytoFlex SRT (Beckman Coulter, CytExpert v.2.4.0.28). For quantification FlowJo software v.10 (BD Biosciences, RRIDSCR_008520) was used.

### Phalloidin staining and quantification

To stain F-actin in LSECs, cells grown on glass plates were fixed with 4% paraformaldehyde (PFA) and washed with PBST (0.2% Triton-X100) three times. Then, 5 µl of stock solution (Alexa Fluor 488 Phalloidin, A12379, Abcam) was diluted with 200 µl PBS for each sample. After 30 min incubation in the dark at room temperature, plates were washed three times with PBST (0.2%) and cell nuclei were stained with 4,6-diamidino-2-phenylindole (DAPI; Sigma Aldrich, D9542) before mounting. The staining was analyzed and imaged using a Zeiss confocal laser microscope (Zeiss LSM 710) operated by ZEN imaging software (Zen v.2.3 SP1 FP3 black). Total cell fluorescence was quantified using Fiji.

### Prediction of transcription factor binding sites

The promotor sequence of the human *SEMA3A* sequence (−1,500 bp upstream, 500 bp downstream of the transcription start site) was loaded and analyzed using CiiiDER^44^ using the following parameters and databases: deficit score 0.15; JASPAR2020_CORE_vertebrates.txt; *Homo_sapiens*.GRCh38.94.glm; and *Homo_sapiens*.GRCh38.dna.primary_assembly.fa. Detected transcription factor binding sites were selected manually using the GUI interface of CiiiDER and the results were exported as an image file.

### Analysis of LSEC fenestration

#### Liver dissociation

To generate a single-cell suspension only consisting of LSECs, the liver dissociation kit (Miltenyi Biotec, 130-105-807) from Miltenyi was utilized. First, PEB solution was prepared (47.5 ml MACS rinsing solution and 2.5 ml BSA/EDTA per animal). This mixture was de-gassed in a magnetic mixer for 15 min. Meanwhile, 500 μl coating solution (PELOBiotech, PB-LU-000-0002-00) was added into wells (24-well plate) and incubated for 30 min at room temperature. Livers were collected and transferred into a gentleMACS C-tube containing the dissociation mix from Miltenyi. The tube was closed and attached onto a sleeve of the gentleMACS Octo Dissociator after which the samples were resuspended and added onto a MACS SmartStrainer (70 μm). Then, 5 ml DMEM were added to the C-tube to collect any remaining cells and applied onto the strainer as well. Last, the Falcon tubes containing the separated cells were centrifuged at 300*g* for 10 min.

#### Magnetic-activated cell sorting

To isolate LSECs from the generated single-cell solution containing all hepatic cell types, the immunomagnetic cell separation system with columns from Miltenyi was used. The last step of the liver dissociation procedure is the centrifugation of the Falcon tubes containing the separated cells. Next, the supernatant was carefully aspirated, the pellet resuspended with 5 ml PEB and then centrifuged again at 300*g* for 10 min. Meanwhile, LS columns for magnetic separation were equilibrated with 3 ml PEB. After centrifugation of cells, the supernatant was removed, the pellet resuspended in 90 μl PEB and 10 μl of magnetic beads coupled to a CD146 antibody (Miltenyi Biotec; 130-092-007) were added. The Falcon tubes, containing the cell suspension and the magnetically labeled CD146 antibodies, were put onto a rotator in the fridge (4 °C) for 15 min. Afterwards, the cells were washed with 1 ml PEB and centrifuged at 300*g* for 10 min, then the supernatant was taken off and the pellet resuspended in 500 μl PEB. This cell suspension was applied onto a column and washed with 3 ml PEB twice. The columns were removed from the magnetic field and with a plunger, the magnetically labeled cells were washed out with 5 ml PEB onto the second column, to which a MACS SmartStrainer (30 μm) was attached. After the column and the MACS SmartStrainer were washed twice with 3 ml PEB, the magnetically labeled cells were flushed out with 5 ml PEB into a fresh 15 ml Falcon tube, which was centrifuged at 900*g* for 3 min. Next, the supernatant was taken off and the pellet was resuspended in pre-warmed EBM-2 medium, which resulted in 60,000 cells per well, and then incubated at 37 °C and 5% CO_2_ for 4 h, after which the cells could be further utilized.

#### Fluorescence-activated cell sorting

For mRNA expression analyses, LSECs of 12-week-old *db/db*, *db/+*, *ob/ob* and wild-type control mice were isolated via MACS (see above) and additionally enriched via FACS, yielding an LSEC purity of >95%. As MACS was only necessary for pre-enrichment, cells were applied to only one MACS column but were washed three times. The magnetically labeled cells were flushed out with 4 ml PEB directly into FACS tubes and centrifuged for 5 min at 300*g*. Next, the supernatant was discarded and cells were resuspended in 300 µl PEB buffer containing 1:50 anti-mouse CD146 PE-conjugated antibody (Miltenyi, 130-118-253). After 15 min of incubation at 4 °C, cells were washed twice with 3 ml PEB buffer and centrifuged at 300*g* for 3 min. Cells were resuspended in 2 ml PEB buffer and up to 200,000 single CD146^+^ LSECs per mouse were sorted at a CytoFLEX SRT (Beckman Coulter).

#### Treatment of mouse LSECs with Semaphorin-3A-Fc

After allowing LSECs to grow for 4 h in EBM-2 medium with supplements, the cells were starved for another hour using EBM-2 medium without supplements. After 1 h, the medium was aspirated and treated with either a control protein (IgG2a-Fc, Recombinant Mouse IgG2a-Fc Protein, R&D Systems, 4460-MG-100) or different concentrations of recombinant semaphorin-3A (SEMA3A-Fc, Recombinant mouse semaphorin-3A Fc Chimera Protein, R&D Systems, 5926-S3-025) reconstituted in PBS; however, the total amount of protein was kept constant. After the cells were incubated at 37 °C and 5% CO_2_ for the desired amount of time, they were fixed in either PFA (4% in PBS) or glutaraldehyde (2% in sodium cacodylate buffer).

#### Antibody and inhibitor treatments of LSECs

After 4 h of incubation, isolated LSECs were treated with different types of NRP1 antibodies (anti-NRP1^SEMA3A^; Genentech^47^, anti-NRP1; R&D Systems, AF566), while anti-NRP1^VEGF^ (Genentech) served as a control^47^. The antibodies were diluted with EBM-2 medium without supplements (to simultaneously starve the cells) at a final concentration of 5 µg ml^−1^. After addition of the antibodies, the cells were incubated at 37 °C and 5% CO_2_ for 1 h.

If LSECs were to be pretreated with the LIMK1 inhibitor LIMKi 3 (Tocris, 4745), they were allowed to grow 4 h and then incubated with LIMKi 3 for 1 h at 37 °C and 5% CO_2_. The inhibitor was diluted to a final concentration of 3 µM in EBM-2 medium without supplements and DMSO with a final concentration of 0.1%. As a control, the cells were treated with EBM-2 medium without supplements with the same concentration of DMSO (0.1%).

#### SEM of mouse LSECs

After treatment, the glass plates were removed from the wells and transferred to a 24-well plate containing 500 μl of glutaraldehyde solution (2% in sodium cacodylate buffer in a total of 2 ml: 160 μl 25% glutaraldehyde (stock) solution + 1,840 μl sodium cacodylate buffer (0.1 M)) per well. The next day, the glutaraldehyde solution was taken off and 500 μl sodium cacodylate buffer (0.1 M) was applied onto each glass plate. Following this, the sodium cacodylate buffer (0.1 M) was taken off and the cells were incubated with 500 μl OsO_4_ solution (4 ml total: 3 ml 0.1 M sodium cacodylate buffer + 1 ml 4% OsO_4_) per well for 30 min. Next, the cells were washed twice with 500 μl of cacodylate buffer for 5 min. Then, 500 μl of 70% ethanol was added into each well and incubated for 5 min. This step was repeated with 80% and 90% ethanol after which the glass plates were transferred into a 24-well plate containing 500 μl of 100% ethanol. Last, the cells were chemically dried using tetramethylsilane (TMS) (ACROS Organics, Thermo Fisher Scientific). The TMS was added into each well, approx. until the volume doubled (1:1 ratio of ethanol to TMS). After 30 min of incubation, TMS was again added until the volume doubled and incubated for 30 min. Thereafter the cells were aspirated and a few drops of TMS were added into each well, just covering the glass plate and incubated for 30 min. After the cells were aspirated, a few drops of TMS were added into each well and the plates were left to dry overnight. The plates were removed and attached onto SEM Specimen Stubs (12.5 mm Ø, 3.2 × 8 mm pin) using double-sided adhesive circles. Using a sputter coater, the plates were coated with a thin layer of gold. After this step, the samples were ready to be examined by SEM. For image acquisition, the Leo 1430 VP SEM, Zeiss FIB-SEM 540 Crossbeam or Zeiss SUPRA 55VP, together with the Zeiss imaging software, were utilized.

#### Manual quantification of fenestrae diameter, frequency and porosity

For the morphologic analysis of LSECs, the images obtained with the Leo 1430 VP were examined using the Fiji imaging-processing package^56^. Analyzed features were the fenestrae frequency (the number of fenestrae per μm^2^), the LSEC porosity (the ratio of fenestrated area to the analyzed cell area) and the fenestrae diameter. First, the scale was set from pixel to μm, to measure all parameters in the intended unit. Next, the cell area was determined, using the polygon selection tool. The outline of the cells was traced and the area was measured in μm^2^. To count the number of fenestrae on the LSEC surface, the Cell Counter Plugin was utilized (plugins → analyze → cell counter → cell counter). For a better resolution, the contrast and brightness were adjusted and the processing tool ‘smooth’ was applied (image → adjust → brightness/contrast, process → smooth). Then, the fenestrae were counted and a copy, where all the fenestrae are flagged, was saved to aid the measuring of the fenestrae diameter. The diameter was measured using the straight-line tool and the measurements were given in μm. All obtained measurements were used to calculate above-mentioned parameters by using equations (1) and (2).

1 $${{\mathrm{Fenestration}}\,{\mathrm{frequency}}\,({\upmu}{{\mathrm{m}}}^{-2})=\frac{{\mathrm{Number}}\,{\mathrm{of}}\,{\mathrm{fenestrae}}}{{\mathrm{Analyzed}}\,{\mathrm{cell}}\,{\mathrm{area}}\,({\upmu}{\mathrm{m}}^{2})}}$$

2 $${{\mathrm{LSEC}}\,{\mathrm{porosity}}=\frac{{\Sigma}\,{\mathrm{Fenestrae}}\,{\mathrm{area}}\,({\upmu}{\mathrm{m}}^{2})}{{\mathrm{Analyzed}}\,{\mathrm{cell}}\,{\mathrm{area}}\,({\upmu}{\mathrm{m}}^{2})}}$$

#### Quantification of fenestrae diameter, frequency and LSEC porosity with machine learning

For the morphologic analysis of LSECs, the images obtained with the Zeiss FIB-SEM 540 Crossbeam or SUPRA 55VP were examined using a deep-learning workflow that is based on the uncertainty-aware variant^78^ of the Contour Proposal Network (CPN)^79^. This model was specifically chosen for its capability to directly predict object contours in biomedical image data, providing an accurate representation of object shapes and sizes. It uses a U-Net architecture^80^ with a ResNeXt-101 encoder^81^. This setup utilized a pretrained network (ginoro_CpnResNeXt101UNet-fbe875f1a3e5ce2c) from the celldetection Python package (https://github.com/FZJ-INM1-BDA/celldetection), designed for multimodal cell segmentation. The model was fine-tuned using manual annotations and applied with an ensemble strategy. Computations were performed on the JUWELS supercomputer^82^.

### G-actin/F-actin in vivo assay biochem kit

Quantification of F-actin and G-actin in primary mouse LSECs was performed using the G-Actin/F-Actin In Vivo Assay Biochem kit from Cytoskeleton (cat. no. BK037). To this end, LSECs were isolated using MACS, incubated for 4 h, starved for 1 h and treated for 1 h with 1 µg ml^−1^ of either SEMA3A-Fc or IgG2a-Fc. The division of F-actin and G-actin was performed according to the description of the kit. Afterwards, both fractions were analyzed using western blotting (antibody used was anti-actin monoclonal antibody (clone 7A8.2.1; cat. no. AAN02-S)). For quantification, a dilution series was used to generate a standard curve.

### Western blotting

For the western blot sample preparation, cells were lysed with radioimmunoprecipitation assay (RIPA) buffer (50 mM Tris-HCl, pH 7.4, Sigma-Aldrich; 150 mM NaCl, Roth; 1 mM EDTA, Ambion; 1 mM Na_3_VO_4_, Sigma-Aldrich; 1 mM NaF, Sigma-Aldrich, 0.25% sodium deoxycholate, AppliChem; 1% IGEPAL, Sigma-Aldrich, in H_2_O plus protease inhibitor, Sigma, 11697498001 and phosphatase inhibitor, Sigma, 4906845001). The lysates were disrupted (Disruptor Genie, Scientific Industries) and centrifuged at 4 °C, 15,700*g* (Centrifuge 5415R, Eppendorf) and the supernatant was collected. Protein concentrations of the samples were determined using a Pierce BCA protein assay kit (Thermo Scientific, 23225) and all samples were diluted to the amount of the sample with the least amount of protein, while containing 20 μg at most. All samples were filled up with water to 30 μl, 10 μl 4× Laemmli sample buffer (180 μl 4× Laemmli stock, 20 μl NaF, 40 μl Protease inhibitor (Roche) and 10 μl β-mercaptoethanol) was added and the samples were incubated at 95 °C for 5 min for protein denaturation. Following, the samples were put on ice for immediate use. A Mini-PROTEAN TGX Stain-Free Protein Gel was loaded with 10–15 μl sample per lane. As a ladder 5 μl of PageRuler Prestained Protein Ladder (Thermo Fisher) was used. Gels were run at 120 V for approximately 40 min. The stain-free gel was immediately activated using UV light for 5 min. After imaging the gel, two ion transfer stacks and the blotting membrane were assembled in the transfer chamber of the Trans-Blot Turbo according to the manufacturer’s instructions. Following the transfer, the blot was imaged using Bio-Rad ChemiDoc MP Imaging software. Then, the blot was blocked in 5% milk in 1× PBST for 1 h.

To visualize cofilin-1 and p-S3-cofilin-1, the blots were incubated for at least 16 h or overnight in the primary antibody at 4 °C on a horizontal shaker (antibodies were p-S3-cofilin-1, Cell Signaling, 3313T, 1:750 dilution; cofilin-1, Cell Signaling, 5175T, 1:750 dilution and GAPDH, Abcam, ab9485, 1:2,500 dilution).

Afterwards the blots were washed three times with 1× TBST for 5 min and then incubated for 1 h with the secondary antibody (antibodies were anti-rabbit IgG, HRP-linked antibody, Jackson Immuno Research, 711-035-152, 1:4,000 dilution; and anti-rabbit IgG, HRP-linked antibody, Invitrogen, G21234, 1:2,000 dilution) on a horizontal shaker at room temperature. They were washed again three times with 1× TBST for 5 min before applying Pierce ECL Western Blotting substrate (Thermo Fisher) onto the membrane to detect specific protein bands. The membrane was incubated in the substrate for 5 min and the ChemiDoc MP and the ImageLab v.4.1 software from Bio-Rad were used to develop and analyze images.

### Luminescent cell viability assay

LSECs were isolated, plated onto white, opaque 96-well cell culture plates, incubated for 4 h and fasted for 1 h, all at 37 °C and 5% CO_2_. Last, they were treated with 0, 0.5 or 2 μg ml^−1^ SEMA3A-Fc for 1 h, while they stayed at 37 °C and 5% CO_2_ for 30 min. Then they were taken out of the incubator and equilibrated at room temperature for another 30 min. ATP measurements were performed using the CellTiter-Glo kit (G7570, Promega) according to the supplier’s instructions. Luminescence was finally measured using the Promega GloMax with the firmware v.4.88.0 and the software v.2.4.1 (emission filter, none; and integration time, 0.3 s).

### Kinase activity profiling

The PamGene assays measure kinase activity in cell and tissue lysates by measuring the phosphorylation of peptide representations of kinase targets/substrates that are immobilized on the PamChip microarrays. The active kinases in the sample lysates will phosphorylate their target on the array. Generic fluorescently labeled antibodies that recognize phosphorylated residues are used to visualize the phosphorylation. We employed both types of PamChip microarrays, the PTK and the STK microarray, with 340 different substrates in total.

To perform kinase activity profiling, mouse LSECs were isolated using MACS and cells were seeded at 1 × 10^6^ cells per well on a six-well plate. After 4 h, cells were starved for 1 h and then treated for 10 min with 1 µg ml^−1^ of either SEMA3A-Fc or IgG2a-Fc. Afterwards, the plate was put on ice, the culture medium was removed and cells were washed with cold PBS. After removal of PBS, the washing step was repeated. Lysis buffer (Halt Phosphatase Inhibitor Cocktail and Halt Protease Inhibitor Cocktail EDTA free, 1:50/1:100 diluted in M-PER Mammalian Extraction Buffer) was added to the cells and cells were collected using a cell scraper. Cells were lysed by pipetting up and down several times over the course of 15 min on ice. Samples were centrifuged for 15 min at 16,000*g* at 4 °C. The lysate was collected and transferred to a clean vial on ice. After snap-freezing in liquid nitrogen, samples were stored at −80 °C until transport to the PamGene facility. The analysis and data processing were performed by PamGene (‘s-Hertogenbosch, Netherlands).

### Liver perfusion for SEM preparation

Liver perfusion and fixation were performed according to the protocol from Cogger et al.^83^. After fixation, the livers were placed onto specimen stubs (12.5 mm Ø, 3.2 × 8 mm pin) using double-sided adhesive circles. Using a sputter coater, the plates were coated with a thin layer of gold in a controlled and even manner. After this step, the samples were ready to be examined by SEM. For image acquisition, the Leo 1430 VP SEM, Zeiss FIB-SEM 540 Crossbeam or Zeiss Supra 55VP, together with the Zeiss imaging software, were utilized.

### Semi-automatic quantification of SEM images of liver sinusoids

SEM images were quantified using Fiji with help of the trainable WEKA Segmentation plugin^56,57^. First, an automatic contrast (‘normalize local contrast’) was calculated for each SEM image and the polygon selection tool of Fiji was used to manually mark the area of interest (sinusoid area) and all non-sinusoid area and gaps were cleared. Next, the fenestrae area and sinusoid cell surface area was identified using a dataset-trained-classifier segmentation (WEKA) algorithm in Fiji^56,57^. The classifier was trained using typical SEM images and stored in a classifier file. The classifier segmentation (WEKA) algorithm led to the generation of probability maps for cell surface area and fenestrae area (Extended Data Fig. 5a). Finally, the maps were used to calculate the overall surface area of the sinusoid and also to quantify fenestrae using the ‘analyze particles’ feature, which returns the area and diameter of each object. Small objects or objects with a low circularity (circularity <0.50) were excluded from the analysis, as fenestrae are expected to be round or oval in shape. The data were transferred to Excel (Microsoft) and the frequency (no. fenestrae per area) and porosity (fenestrae area per area analyzed) was calculated.

### Histology and Oil Red O staining of liver sections

Hepatic TG content was quantified by staining liver cryosections with ORO. To this end, freshly isolated livers were frozen in OCT medium (TissueTek) using dry ice. Liver cryosections (12 µm) were stained in filtered ORO working-solution (24 parts stock solution (300 mg ORO, Sigma-Aldrich, O-9755 in 100 ml 2-propanol) + 16 parts demineralized water). Sections were rinsed briefly in demineralized water and washed for 10 min in running tap water and embedded using Fluoroshield (Sigma-Aldrich, F6182). An Eclipse Ti-S microscope (Nikon) and a DS-2Mv camera operated by NIS-Elements software (Nikon) were used for imaging. Fiji^56^ was used to measure sections and the lipid droplet area. Finally, the ratio of stained/section area was calculated for each image. For paraffin sections, H&E and Elastica van Gieson staining was performed at the Histopathology Diagnostic Laboratory of the Institute of Pathology using an autostainer (TissueTek Prisma) according to standardized protocols. For H&E-staining of cryosections, sections were stained with Mayer’s hematoxylin (Sigma, MHS 16) and eosin Y solution (Sigma, E4282), dehydrated and embedded in Entellan (Sigma, 1.07961.0100). For PSR staining, cryosections were stained for 60 min in PSR solution (0.1 g Sirius Red, Direktrot 80; Sigma 365548 in 100 ml saturated aqueous picric acid), dehydrated and prepared for microscopy.

### Immunohistochemical staining of cryosections

Immunofluorescence staining was performed with liver cryosections (12 µm) of male C57BL/6J mice that were fixed with 4% PFA. Sections were treated with blocking solution (10% normal donkey serum, 2% BSA, PBS and 0.2% Triton-X100) for 1 h and incubated overnight with primary antibodies in blocking solution using rabbit anti-LYVE1 (Abcam, AB14917, lot GR320055-2); goat anti-NRP1 (Research and Development, AF566, lot ETH0612091) or isotype control goat IgG (Santa Cruz, SC2028, lot A2913). After washing the sections in PBS (0.2% Triton-X100) for 3 × 5 min, sections were incubated for 1 h at room temperature with secondary antibodies (donkey anti-goat Alexa 555, Invitrogen, A21432, lot 1818686; donkey anti-rabbit Alexa 488, Invitrogen, A21206) and DAPI (1 µg ml^−1^; Sigma-Aldrich, D9542). Finally, sections were washed with PBS (0.2% Triton-X100) for 2 × 5 min and embedded using Fluoroshield medium (Sigma-Aldrich, F6182) and a coverslip. Staining was analyzed and imaged using a Zeiss confocal laser microscope (Zeiss LSM 710) operated by ZEN imaging software (Zeiss). Images were analyzed using Fiji^56^.

### Serum parameters

To measure TGs, ALT, AST, total cholesterol (Chol) and high-density lipoprotein (HDL) cholesterol in the serum of fasting mice, Kenshin-2 Spotchem 4430 test stripes were used in combination with the SPOTCHEM EZ SP-4430. Values <15 (n.d.) were defined as 15. The samples were measured according to the manufacturer’s description. NEFA was measured using the NEFA-HR(2) Assay (FUJIFILM Wako Chemicals) and insulin was measured using an ultra-sensitive rat insulin ELISA (Crystal Chem, cat. no. 90060). HOMA-IR was calculated as (insulin × glucose (ng ml^−1^ × ml dl^−1^)/405) and Adipo-IR (FFA × insulin (mmol l^−1 ^pmol^−1^) and expressed as percentage of control.

### Glucose tolerance test

Mice were subjected to an overnight fast before undergoing GTTs. In the test, glucose (1 mg g^−1^ body weight) was intraperitoneally injected and blood glucose levels were assessed by obtaining blood samples from the tail tip. Using a Monometer Futura glucometer (MedNet), blood glucose concentrations were measured twice at each time point. To measure plasma insulin concentrations, small amounts of blood were collected from the tail tip using EDTA-coated tubes, followed by plasma preparation through a 10-min centrifugation at 2,000*g*. Insulin concentrations were subsequently measured using an ultra-sensitive rat insulin ELISA (Crystal Chem).

### VLDL secretion assay

Mice were weighed and subjected to a 4-h fast before undergoing the VLDL secretion test. In the test, 0.5 g kg^−1^ body weight of WR1339 (Sigma, T8761) was intraperitoneally injected and blood was collected from the tail tip pre-injection and after 1, 2, 4 and 6 h after Triton WR1339 injection using EDTA-coated tubes, followed by plasma preparation through a 10-min centrifugation at 2,000*g*. TGs were measured using a LabAssay Triglyceride kit (FUJIFILM Wako Chemicals Europe) according to the suppliers’ instructions.

### Metabolic cage analysis

Metabolic cages (PhenoMaster, TSE-System) were employed to measure parameters such as physical activity and food intake. Following an adaptation phase in the cages used for measurements, activity and metabolic parameters were continuously monitored. Infrared sensor frames recorded activity, and a control unit identified interruptions in the infrared sensors. Relevant data were registered by a computer using the PhenoMaster software from TSE Systems. Body weight, food and water intake, carbon dioxide production, oxygen consumption and cage temperature were quantified through integrated sensors. The respiratory exchange ratio and energy consumption were calculated by the PhenoMaster software and normalized to body weight and lean mass for relevant parameters. Lean and fat mass was measured using an NMR Analyzer (Minispec, Bruker).

### Statistical analysis

All imaging analyses were performed under blinded conditions. Data were gathered and processed using Excel (Microsoft) and then transferred to GraphPad Prism (v.9.4.0) to generate all graphs. All data points were plotted individually together with the mean and s.e.m. Statistical analysis was performed using GraphPad Prism (v.9.4.0). No statistical outlier tests were applied. If necessary, samples/data were solely removed based on technical issues during the experiments. A two-tailed unequal variance *t*-test (Welch’s test) was used to determine statistical significance between two independent experimental groups. A pairwise Student’s *t*-test was performed to determine statistical significance for samples of the same mouse. In case of multiple *t*-tests in the same analysis in Figs. 1a,b and 2a,b, a multiple two-tailed *t*-test (paired or unpaired) with a two-stage step-up method according to Benjamini, Krieger and Yekutieli^84^ was used to correct for multiple comparisons and to detect significant discoveries. For more than two experimental groups with one or two factors, a one- or two-way analysis of variance (ANOVA) (with or without repeated measurements) was conducted, followed by a Dunnett’s, Tukey’s or Sidak’s post hoc test. Information about the performed statistical tests and samples sizes is indicated in the figure legends. Only *P* and *q* values <0.05 are shown in the figures.

### Reporting summary

Further information on research design is available in the Nature Portfolio Reporting Summary linked to this article.

### Supplementary information

Supplementary Information Supplementary Figs. 1–4.

Reporting Summary

Supplementary Tables 1–4 Table 1 Primer sequences. Table 2 UKA PamGene data. Table 3 MTvC PamGene data. Table 4 Statistical source data for Supplementary Fig. 3.

### Source data

Source Data Fig. 1 Unprocessed agarose gels.

Source Data Fig. 3 Unprocessed actin western blots and unprocessed SEM images.

Source Data Fig. 4 Unprocessed SEM images/uncropped kinase tree.

Source Data Fig. 5 Unprocessed cofilin western blots and unprocessed SEM images.

Source Data Extended Data Fig. 8 Unprocessed agarose gel.

Source Data Figs. 1–8 and Extended Data Figs. 1–8 Statistical source data of main and extended data figures.

## Data Availability

Data from LSEC kinase activity screening, source data (blots and gels) and statistical Source data are provided with this paper. All additional data are available upon request from the corresponding author.

## Code availability

Original plugin code and scripts for LSECs are available on GitHub at https://github.com/sybal100/S3A_NCR.
