# Supplementary material for: Semaphorin-3A regulates liver sinusoidal endothelial cell porosity and promotes hepatic steatosis
Source: Nat Cardiovasc Res. 2024 Jun 14;3(6):734–53. doi: 10.1038/s44161-024-00487-z (PMC11358038; doi:10.1038/s44161-024-00487-z)
Supplement: Supplementary file 1 — Supplementary Figs. 1–4. [file 44161_2024_487_MOESM1_ESM.pdf]

nature cardiovascular research

Supplementary information https://doi.org/10.1038/s44161-024-00487-z

Semaphorin-3A regulates liver sinusoidal endothelial cell porosity and promotes hepatic steatosis

In the format provided by the authors and unedited

Supplementary Information

**Figure**	**Title**
Supplementary Information Fig. 1.	FACS-gating strategy for isolation of CD146 positive LSEC.
Supplementary Information Fig. 2.	Analysis of viability of primary human LSEC upon treatment with fatty acids.
Supplementary Information Fig. 3	F-actin staining of human LSEC
Supplementary Information Fig. 4.	Full list of predicted transcription factor binding sites in the *SEMA3A* promoter.
Supplementary Information Table 1.	Tab. 1. Primer sequences<br>Tab. 2. UKA PamGene data<br>Tab. 3. MTvC PamGene data<br>Tab. 4. Statistical source data for Supplementary figure 3
Ethical certificates	Statement of the authors

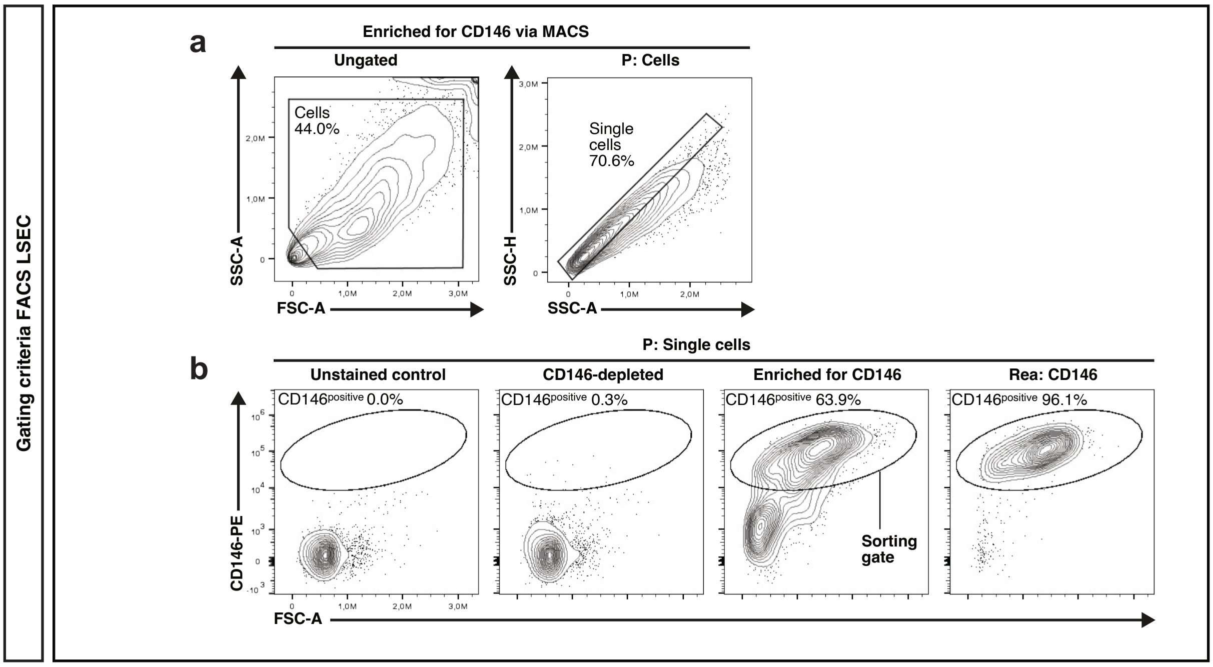

Supplementary Fig. 1. FACS-gating strategy for isolation of CD146-positive LSEC. a, FSC-A *versus* SSC-A of dispersed liver cells that were pre-enriched for CD146-positive LSEC via MACS. "Cells"-gate was used for exclusion of cell debris (left panel). SSC-A *versus* SSC-H of the "Cells"-population. "Single cells"-gate was used for exclusion of cell duplets (right panel). **b**, FSC-A *versus* CD146-PE of the "Single cells"-population. Unstained and CD146-depleted cells (MACS negative fraction) were used as controls (first and second panels). Cells enriched for CD146 were used for sorting of CD146-positive LSEC (third panel). Reanalysis of sorted cells was performed to assess purity (last panel). P: Population; Rea: Reanalysis (after sorting).

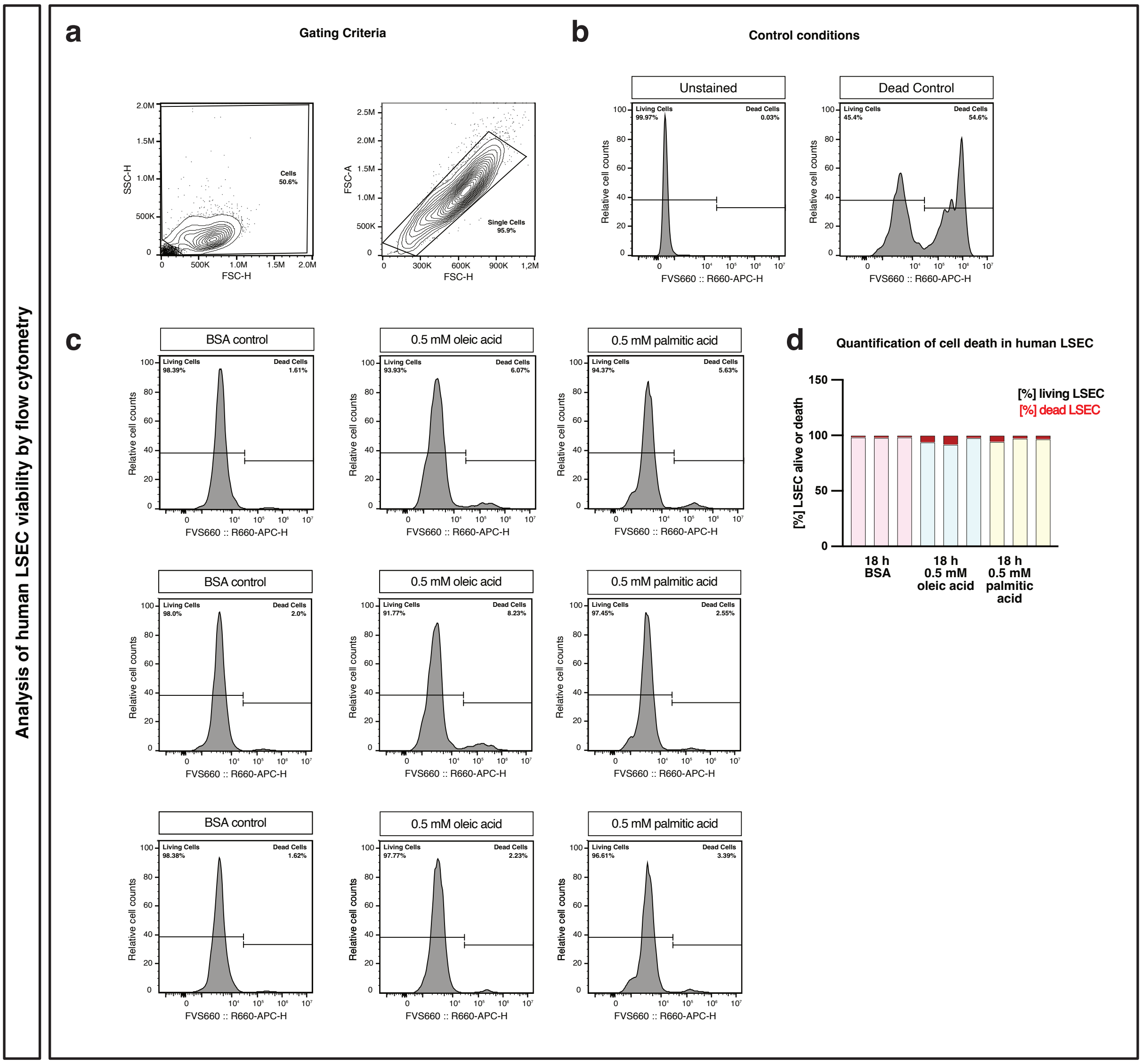

Supplementary Fig. 2. Analysis of viability of primary human LSEC upon treatment with fatty acids. Human LSEC viability measured by flow cytometry. **a**, Gating criteria of "Cells" (excluding cell debris), utilizing an FSC-H *versus* SSC-H contour plot, and "Single Cells" (excluding cell duplets), utilizing an FSC-H *versus* FSC-A contour plot of the "Cells" population, as well as **b**, Viability staining, using an "Unstained" control and a "Dead Control" (human LSEC incubated at 60°C for 5 minutes) histogram of the FVS660 channel. **c**, Viability staining of LSEC treated with BSA (control), 0.5 mM oleic acid or 0.5 mM palmitic acid are shown as histograms of the FVS660 channel (n = 3 wells per condition). **d**, Quantification of dead cells is shown as a stacked bar graph. LSEC death, as determined by FVS660 staining, showing the percentage of FVS660-negative (living) cells and FVS660-positive (dead) cells.

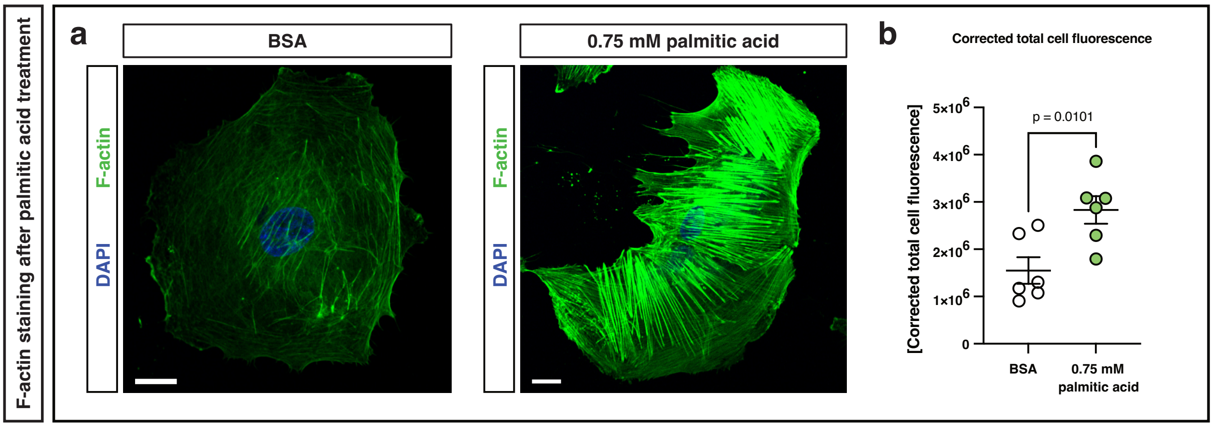

Supplementary Fig. 3. F-actin staining of human LSEC. a, Phalloidin staining of human LSEC (male donor: QC-12B15F11) treated with 0.75 mM palmitic acid or BSA for 24 h, scale bars = 20 µm. Brightness and contrast have been adjusted to enhance visibility. In all graphs individual data points and mean ± SEM are presented. b, Densiometric quantification of actin fluorescence. A two-tailed unequal variances *t*-test was used to test for statistical significance. N = 6 images of a cell culture experiment.

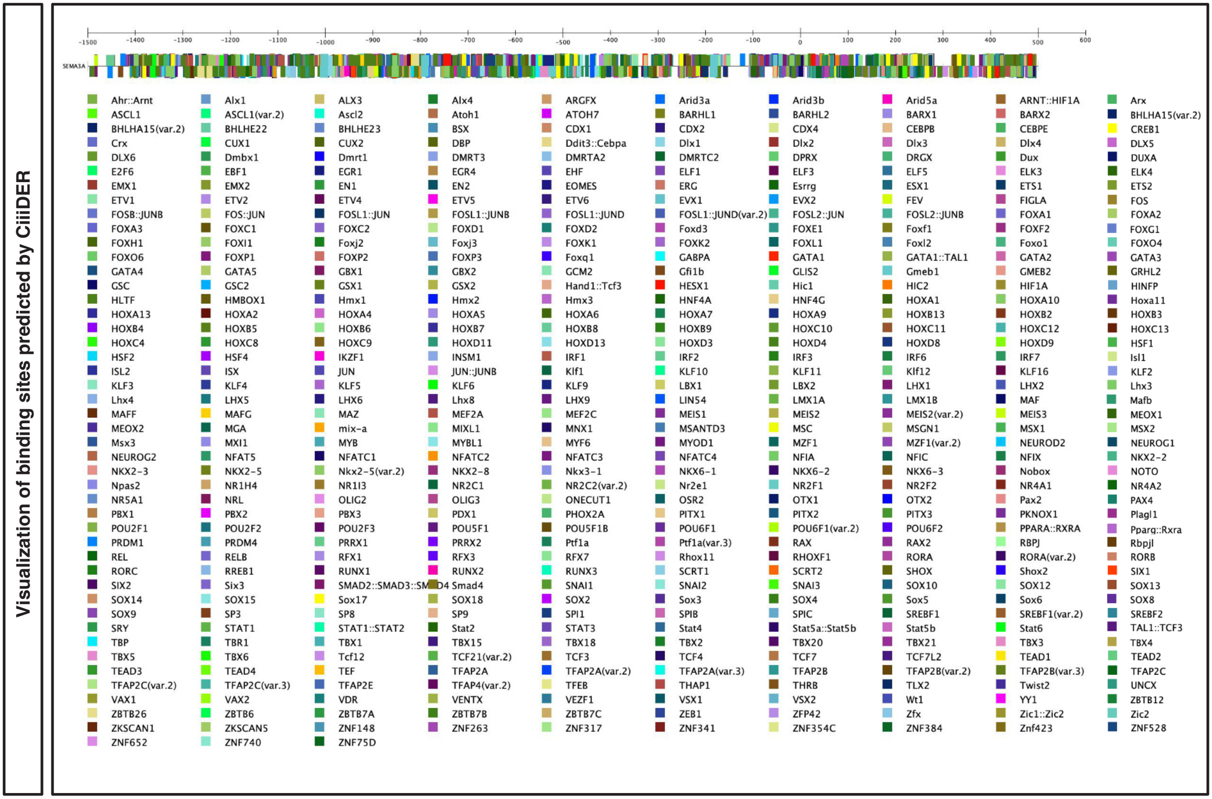

Supplementary Fig. 4. Full list of predicted transcription factor binding sites in the human *SEMA3A* promoter. List of transcription factor (TF) binding sites in the promotor of the human *SEMA3A* gene (1,500 bp upstream, 500 bp downstream of ATG) predicted by CiiiDER.

Ethical certificates:

Hereby, the authors certify that they obtained statements from the companies ThermoFisher Scientific, KaLy-Cell and PELOBiotech, confirming that all human cells purchased were obtained with the consent of each donor.

Eckhard Lammert
(on behalf of all authors)
