# Supplementary material for: Semaphorin-3A regulates liver sinusoidal endothelial cell porosity and promotes hepatic steatosis
Source: Nat Cardiovasc Res. 2024 Jun 14;3(6):734–53. doi: 10.1038/s44161-024-00487-z (PMC11358038; doi:10.1038/s44161-024-00487-z)
Supplement: Supplementary file 2 — Reporting Summary [file 44161_2024_487_MOESM2_ESM.pdf]

nature portfolio

Corresponding author(s): Eckhard Lammert

Last updated by author(s): Apr 30, 2024

Reporting Summary

Nature Portfolio wishes to improve the reproducibility of the work that we publish. This form provides structure for consistency and transparency in reporting. For further information on Nature Portfolio policies, see our Editorial Policies and the Editorial Policy Checklist.

Statistics

For all statistical analyses, confirm that the following items are present in the figure legend, table legend, main text, or Methods section.

n/a	Confirmed	
☐	☒	The exact sample size (*n*) for each experimental group/condition, given as a discrete number and unit of measurement
☐	☒	A statement on whether measurements were taken from distinct samples or whether the same sample was measured repeatedly
☐	☒	The statistical test(s) used AND whether they are one- or two-sided<br>*Only common tests should be described solely by name; describe more complex techniques in the Methods section.*
☒	☐	A description of all covariates tested
☐	☒	A description of any assumptions or corrections, such as tests of normality and adjustment for multiple comparisons
☐	☒	A full description of the statistical parameters including central tendency (e.g. means) or other basic estimates (e.g. regression coefficient) AND variation (e.g. standard deviation) or associated estimates of uncertainty (e.g. confidence intervals)
☐	☒	For null hypothesis testing, the test statistic (e.g. *F*, *t*, *r*) with confidence intervals, effect sizes, degrees of freedom and *P* value noted<br>*Give P values as exact values whenever suitable.*
☒	☐	For Bayesian analysis, information on the choice of priors and Markov chain Monte Carlo settings
☒	☐	For hierarchical and complex designs, identification of the appropriate level for tests and full reporting of outcomes
☐	☒	Estimates of effect sizes (e.g. Cohen's *d*, Pearson's *r*), indicating how they were calculated

Our web collection on statistics for biologists contains articles on many of the points above.

Software and code

Policy information about availability of computer code

Data collection	ZEN Microscopy Software, Zeiss; NIS-Elements software (Nikon, Tokyo, Japan), and software controlling / corresponding to the Leo 1430 VP SEM, Zeiss FIB-SEM 540 Crossbeam and Zeiss SUPRA 55VP were used for image acquisition. TSE PhenoMaster Software was used to control the PhenoMaster hardware. Image Lab Touch Software Version 2.3.0.07 (BioRad) was used for Western Blots, Glow Max Discover 3.2.3 was used for ATP measurements, and MX Pro Mx3000 P Version 4.10. (Stratagene) and Quantstudio Design & Analysis Software v 1.5.1 was used for RT-qPCR.
Data analysis	Data was gathered and processed using Excel (Microsoft). Statistical analysis was performed using Graphpad Prism (9.4.0). ImageLab 4.1 software from Bio-Rad was used to quantify Western Blot images, Fiji (ImageJ) was used to analyze image data.

For manuscripts utilizing custom algorithms or software that are central to the research but not yet described in published literature, software must be made available to editors and reviewers. We strongly encourage code deposition in a community repository (e.g. GitHub). See the Nature Portfolio guidelines for submitting code & software for further information.

Data

Policy information about availability of data

All manuscripts must include a data availability statement. This statement should provide the following information, where applicable:

- Accession codes, unique identifiers, or web links for publicly available datasets
- A description of any restrictions on data availability
- For clinical datasets or third party data, please ensure that the statement adheres to our policy

Data from the LSEC kinase activity screening, source data (blots and gels) and statistical source data are provided with this paper. All additional data is available upon reasonable request from the corresponding author.

Human research participants

Policy information about studies involving human research participants and Sex and Gender in Research.

Reporting on sex and gender	N/A
Population characteristics	N/A
Recruitment	N/A
Ethics oversight	N/A

Note that full information on the approval of the study protocol must also be provided in the manuscript.

Field-specific reporting

Please select the one below that is the best fit for your research. If you are not sure, read the appropriate sections before making your selection.

☒ Life sciences ☐ Behavioural & social sciences ☐ Ecological, evolutionary & environmental sciences

For a reference copy of the document with all sections, see nature.com/documents/nr-reporting-summary-flat.pdf

Life sciences study design

All studies must disclose on these points even when the disclosure is negative.

Sample size	G*power was used to calculate animal sample sizes for animal experiments. No sample size calculation was performed for in vitro experiments. The required number of samples for each experiment was determined by referring to past experiments, conducting comparative research, or considering limitations such as the availability of cells (human primary LSEC and hepatocytes).
Data exclusions	No statistical outlier tests were applied. If necessary, samples/data was solely removed based on technical issues during the experiments, i.e. samples, which had concentrations outside the range of the actin standard were excluded. Values of RT-qPCR reactions leading to faulty dissociation curves (> 2 peaks) were excluded from further analysis. SEM images with insufficient (blurry) quality were excluded from the analysis. Mice that experienced weight loss during the final days of the experiment were excluded from subsequent analysis (iECwt, n =1). One mouse was excluded from further glucose measurements, since not the full amount of glucose was accidentally injected (Extended data Figure 6o, n=1).
Replication	The increase of Sema3a expression in the liver/LSEC was observed in three different mouse models with hepatic steatosis, i.e., db/db, ob/ob and HFD-fed vs. their respective controls (Fig. 1 c, d, e). The increase of SEMA3A expression in human LSEC by palmitic acid was replicated in two different donors (male donor, Fig. 2a; female donor, Extended Data Fig. 2a). The reduction of porosity and fenestrae frequency in male mouse LSEC after SEMA3A-Fc treatment was replicated in two independent experiments (Fig. 3d-f and Fig. 5d-f). Body weight reduction was reproducibly observed in two different Sema3a KO mouse models: (1) male chow-fed and HFD-fed Sema3A +/- vs. wt (Extended data Fig. 4b, 6b) and (2) male HFD-fed iECSema3A vs. control mice (Fig.7b, Extended data Fig. 8b). Reduced intrahepatic lipid content in association with Sema3a gene deletion was reproduced in three different Sema3a KO mouse models: 1. Male chow-fed Sema3A +/- vs. wt (Extended Data Fig. 4e,f) and HFD-fed Sema3A +/ vs. wt (Extended data Fig. 6e,f), (2) male HFD-fed VEGFR2-Cre Sema3a fl/fl vs. VEGFR2-Cre Sema3a fl/+ mice (data not shown) and (3) male HFD-fed iECSema3A vs. control (Fig.7e,f). Reduction of hepatic Pparg2 expression was observed in two different Sema3a KO models, (1) male HFD-fed Sema3A +/ vs. wt (Extended Data Fig. 6n) and (2) HFD-fed iECSema3A vs. iECwt (Extended data Fig. 8e).
Randomization	No explicit randomization was performed. For mouse experiments, individual genotypes assigned the mice to experimental groups. Within each genotype, we did not need to further divide the mice into experimental groups. Individual wells containing LSEC in cell culture were not explicitly randomized, given the assumption of uniformity among the cells, either because they were isolated from mice or from the same donor.

Blinding

Microscopy images were collected in a blinded and randomized manner and also analyzed in a blinded or automated (unbiased) way. Investigators were not blinded to mouse genotypes (group allocation) during the experiment nor were they blinded to allocation/data collection and analysis of other cell culture work.

Reporting for specific materials, systems and methods

We require information from authors about some types of materials, experimental systems and methods used in many studies. Here, indicate whether each material, system or method listed is relevant to your study. If you are not sure if a list item applies to your research, read the appropriate section before selecting a response.

Materials & experimental systems

n/a	Involved in the study
☐	☒ Antibodies
☐	☒ Eukaryotic cell lines
☒	☐ Palaeontology and archaeology
☐	☒ Animals and other organisms
☒	☐ Clinical data
☒	☐ Dual use research of concern

Methods

n/a	Involved in the study
☒	☐ ChIP-seq
☐	☒ Flow cytometry
☒	☐ MRI-based neuroimaging

Antibodies

Antibodies used

Goat anti rat/mouse NRP1; R and D Systems; Cat# AF566, RRID:AB_355445, Immunohistochemistry (1:50), receptor blocking (5 µg/ml)
Rabbit polyclonal cofilin; Cell Signaling; Cat# 3318, RRID:AB_2080595, 1:750
Rabbit polyclonal phospho-cofilin; Cell Signaling; Cat#3313T RRID: AB_330238, 1:750
Rabbit polyclonal GAPDH, Abcam, Cat#ab9485, RRID:AB_307275, 1:2500
Donkey anti-rabbit IgG HRP; Jackson ImmunoResearch; Cat# 711-035-152, RRID:AB_10015282, 1:4000
Goat anti-rabbit IgG-HRP, Invitrogen/Thermo Fisher Scientific Cat# G-21234, RRID:AB_2536530, 1:2000
Rabbit anti-LYVE1; Abcam; Cat# ab14917, RRID:AB_301509, 1:100
Normal goat IgG; Santa Cruz Biotechnology Cat# sc-2028, RRID:AB_737167: 1:100
Donkey anti-goat; Alexa555 Thermo Fisher Scientific/Invitrogene Cat# A-21432, RRID:AB_2535853, 1:300
Donkey anti-rabbit Alexa488 Thermo Fisher Scientific/Invitrogene # A-21206, RRID:AB_2535792, 1:300
Goat anti NRP1(1A) Genentech Pan et al., 2007, 5 µg/ml
Goat anti NRP1(1B) Genentech Pan et al., 2007, 5 µg/ml
Anti-mouse CD146; Miltenyi Biotec; Cat# 130-118-253, RRID:AB_2751473, 1:50
Anti-mouse CD146; Miltenyi Biotec; Cat# 130-092-007, RRID:AB_2751473, 1:10
Mouse Anti-Actin MAb (clone 7A8.2.1; Cytoskeleton Cat# AAN02, RRID:AB_2884962)

Validation

Antibodies were validated by the suppliers or producers as follows:
Goat anti rat/mouse NRP1; R and D Systems; Cat# AF566; WB, Immunohistochemistry; FlowCytometry, Blockade of Receptor ligand Interaction
Rabbit polyclonal cofilin; Cell Signaling; Cat# 3318; WB-Western
Rabbit polyclonal phospho-cofilin; Cell Signaling; Cat#3313T; Western Blot, IF
Rabbit polyclonal GAPDH, Abcam, Cat#ab9485, 1:2500; IHC-P, WB, ICC/IF
Donkey anti-rabbit IgG HRP; Jackson ImmunoResearch; Cat# 711-035-152; Western Blot, IHC, ELISA
Normal goat IgG; Santa Cruz Biotechnology Cat# sc-2028, RRID:AB_737167, WB, IF, IHC, FlowCytometry
Goat anti-rabbit IgG-HRP, Invitrogen/Thermo Fisher Scientific Cat# G-21234, RRID:AB_2536530. IP, WB, ELISA
Rabbit anti-LYVE1; Abcam; Cat# ab14917; IHC-P, ICC/IF
Donkey anti-goat; Alexa555 Thermo Fisher Scientific/Invitrogen Cat# A-21432; Immunohistochemistry/IF/ICC
Donkey anti-rabbit Alexa488 Thermo Fisher Scientific/Invitrogen # A-21206; Immunohistochemistry/IF/ICC/FlowCytometry
Anti-mouse CD146; Miltenyi Biotec; Cat# 130-118-253, Flow cytometry, MICS, IF, IHC
Anti-mouse CD146; Miltenyi Biotec; Cat# 130-092-007, MACS
Goat anti NRP1(1A) Genentech Pan et al., 2007; Blockade of Receptor ligand Interaction
Goat anti NRP1(1B) Genentech Pan et al., 2007; Blockade of Receptor ligand Interaction
Mouse Anti-Actin MAb (clone 7A8.2.1; Cytoskeleton Cat# AAN02, RRID:AB_2884962); WB, IF

Eukaryotic cell lines

Policy information about cell lines and Sex and Gender in Research

Cell line source(s)

HepG2 cells were purchased from ATCC (ATCC®HB-8065TM). Culture conditions are stated in the Methods section.
HepG2 is a cell line that was isolated from a hepatocellular carcinoma from a 15-year-old male with liver cancer.

Authentication

HepG2 cells were not authenticated.

Mycoplasma contamination

HepG2 cells were mycoplasm negative (Mycoplasma check was performed by Eurofins).

Commonly misidentified lines (See ICLAC register)	No commonly misidentified cell line were used.

Animals and other research organisms

Policy information about studies involving animals; ARRIVE guidelines recommended for reporting animal research, and Sex and Gender in Research

Laboratory animals	Male 9-11 week-old C57BL/6J mice (Janvier, France), male 14 week-old C57BL/6N, 10- and 12-week-old male db/db.BKS (BKS.Cg-Dock7m +/+ LeprdbJ, JAX #000642), 12-week-old male ob/ob.B6 (B6.Cg-Lepob/J; Jackson Laboratories, USA/JAX #000632) and control mice were used for LSEC isolations and gene expression studies. Male heterozygous Sema3a knockout mice (C57BL/6N background, up to 38 weeks old), male Cdh5-CreERT2 (25 weeks old), and Cdh5-CreERT2 x Sema3a fl/fl (backcrossed to C57BL/6J, 25 week old) were used to study sinusoidal porosity, hepatic lipid content and metabolic parameters.
Wild animals	This study did not involve wild animals.
Reporting on sex	Male mice have been included in this study.
Field-collected samples	This study did not involve samples collected from the field.
Ethics oversight	All animal experiments were approved by the local Animal Ethics Committee of the Landesamt für Natur, Umwelt und Verbraucherschutz Nordrhein-Westfalen (LANUV North Rhine-Westphalia, Germany), and conducted according to the German Animal Protection Laws.

Note that full information on the approval of the study protocol must also be provided in the manuscript.

Flow Cytometry

Plots

Confirm that:

☒ The axis labels state the marker and fluorochrome used (e.g. CD4-FITC).

☒ The axis scales are clearly visible. Include numbers along axes only for bottom left plot of group (a 'group' is an analysis of identical markers).

☒ All plots are contour plots with outliers or pseudocolor plots.

☒ A numerical value for number of cells or percentage (with statistics) is provided.

Methodology

Sample preparation	Mouse LSEC: LSEC of 12-week-old db/db, db/+, ob/ob and wildtype control mice were isolated via MACS and additionally enriched via FACS. The magnetically labelled cells were flushed out with 4 ml PEB directly into FACS tubes and centrifuged for 5 min at 300 x g. Next, supernatant was discarded, and cells were resuspended in 300 µl PEB buffer containing 1/50 anti-mouse CD146 PE-conjugated antibody (Miltenyi 130-118-253). After 15 min incubation at 4°C, cells were washed twice with 3 ml PEB buffer and centrifuged at 300 x g for 3 min. Cells were resuspended in 2 ml PEB buffer and up to 200,000 single CD146 positive LSEC per mouse were sorted at a CytoFLEX SRT (Beckman Coulter).<br><br>Human LSEC: The medium was collected, and the adherent cells were detached by trypsinization and transferred into FACS tubes (Falcon, 352052). FACS tubes were centrifuged (400 x g, 5 min) and cells were washed with PBS (Gibco, 10010-015). The centrifugation step was repeated and FVS660 (BD Biosciences, 564405, 1/1000) diluted in PBS was added for 15 min at RT in the dark. Cells were washed with PBS and centrifuged for 3 min at 400 x g twice. Cell pellet was resuspended in PBS and FVS660 positive (FVS660+, dead cells) and FVS660 negative (FVS660-, living cells) were determined using CytoFlex S (Beckman Coulter, CytExpert Version 2.4.0.28). For quantification FlowJo software version 10 (BD Biosciences, RRID:SCR_008520) was used.
Instrument	CytoFlex S Flow Cytometer (Beckman Coulter, BE35105)
Software	Data was acquired using CytExpert 2.4.0.28 and analyzed using FlowJo V10.10.0
Cell population abundance	A total of 10,000 events were acquired for each sample.
Gating strategy	Cells were gated from debris using an FSC-H/SSC-H contour plot. Subsequently, single cells were gated from duplets by gating the main population of an FSC-H/FSC-A contour plot. The histogram plots of the FVS660 (APC channel) staining were created from the single cell gate.

☒ Tick this box to confirm that a figure exemplifying the gating strategy is provided in the Supplementary Information.
