# Supplementary figures and images for: Semaphorin-3A regulates liver sinusoidal endothelial cell porosity and promotes hepatic steatosis
Source: Nat Cardiovasc Res. 2024 Jun 14;3(6):734–53. doi: 10.1038/s44161-024-00487-z (PMC11358038; doi:10.1038/s44161-024-00487-z)

Unprocessed agarose gels used in Fig 1i

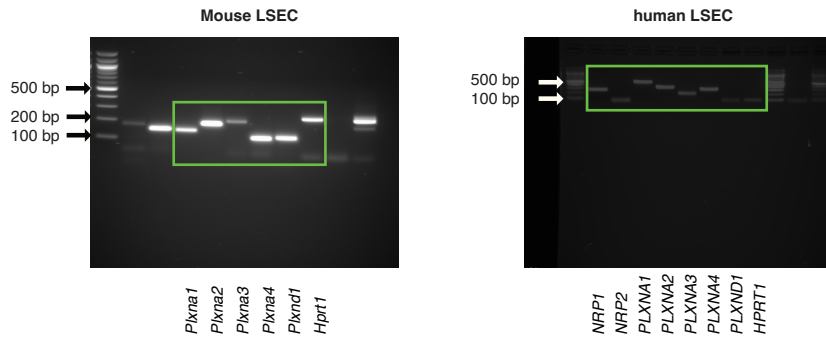

Supplement: Supplementary file 4 — Unprocessed agarose gels. [file 44161_2024_487_MOESM4_ESM.pdf]

Figure 3b - F-/G-actin western blot after SEMA3A-Fc treatment

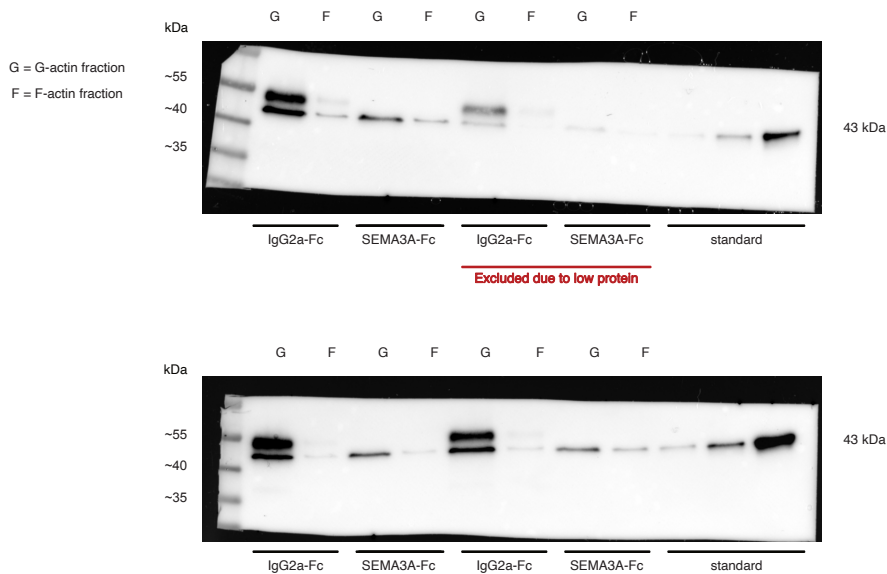

Figure 3c - SEM images

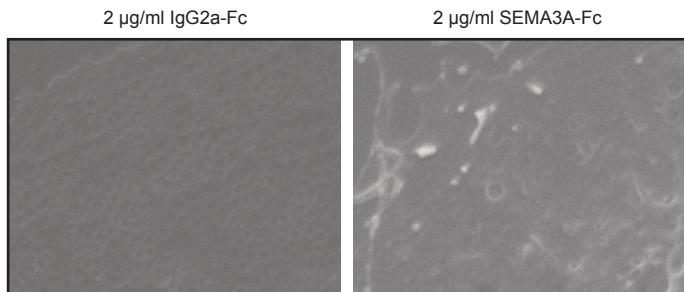

Supplement: Supplementary file 5 — Unprocessed actin western blots and unprocessed SEM images. [file 44161_2024_487_MOESM5_ESM.pdf]

**Figure 4b - SEM images**

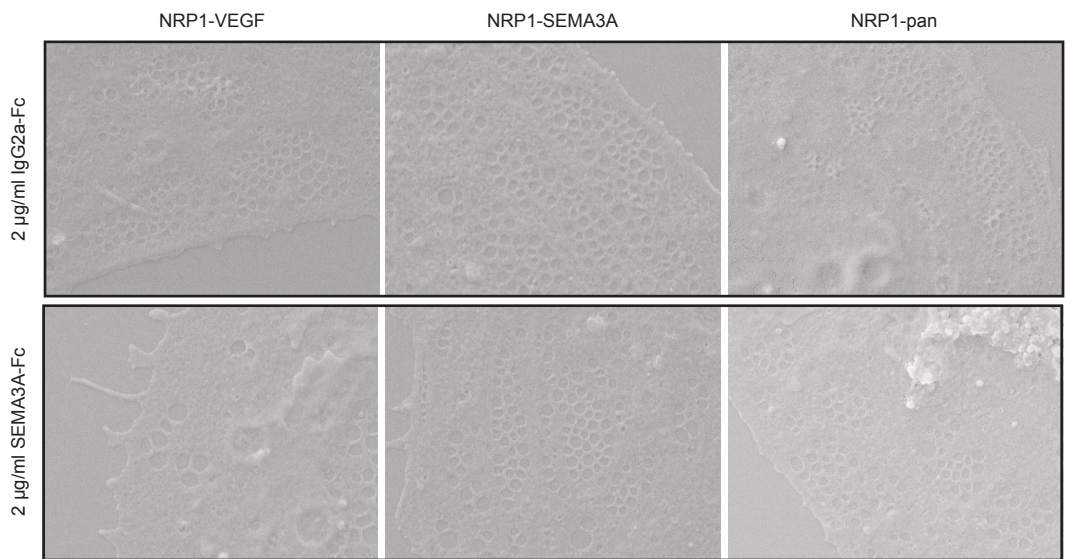

**Figure 4f - Uncropped Kinome Tree**

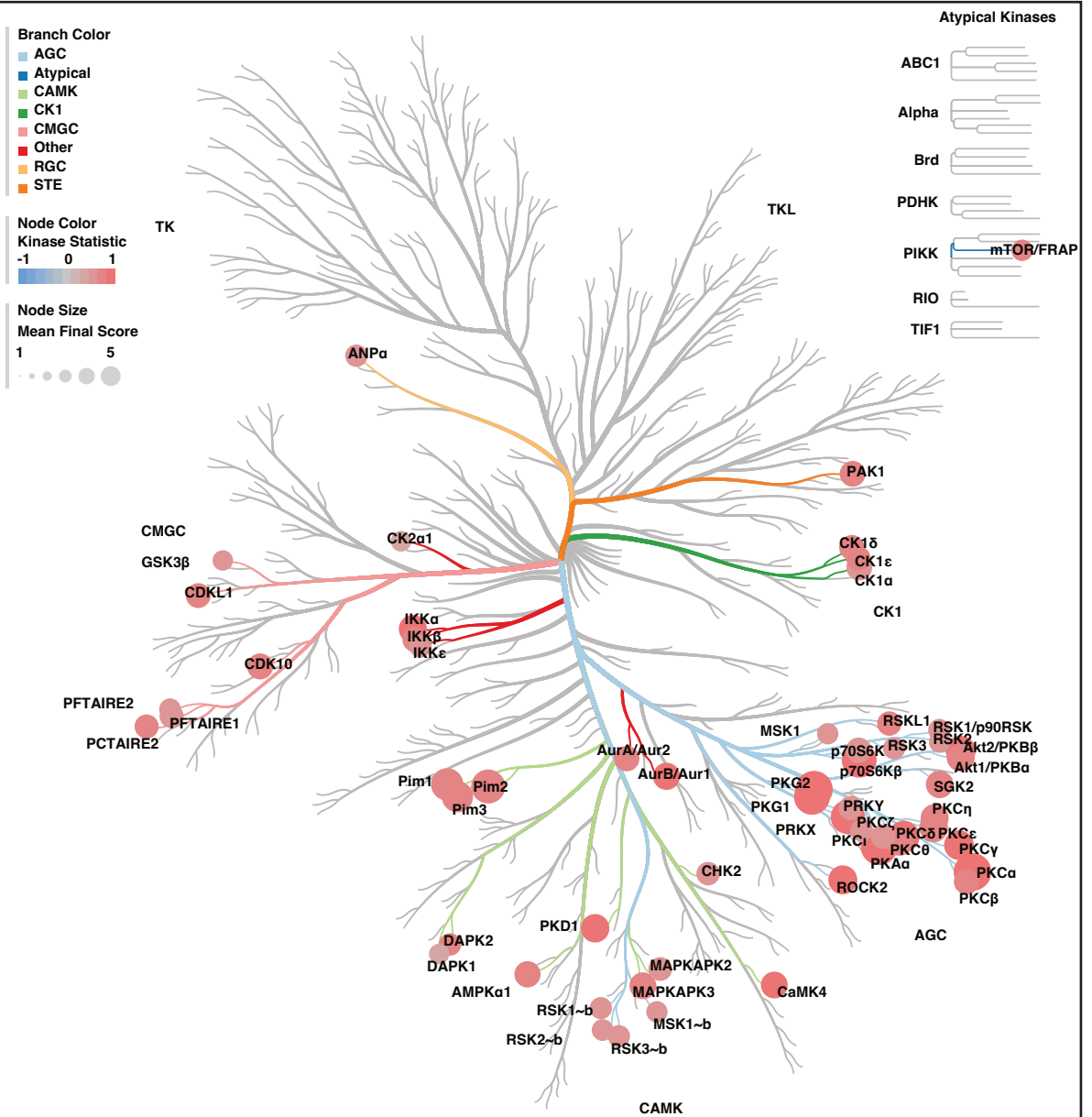

Supplement: Supplementary file 6 — Unprocessed SEM images/uncropped kinase tree. [file 44161_2024_487_MOESM6_ESM.pdf]

Unprocessed agarose gel used to quantify recombination of floxed *Sema3a* alleles

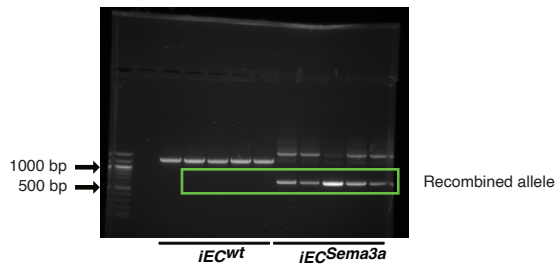

Supplement: Supplementary file 8 — Unprocessed agarose gel. [file 44161_2024_487_MOESM8_ESM.pdf]
