# Supplementary material for: Semaphorin-3A regulates liver sinusoidal endothelial cell porosity and promotes hepatic steatosis
Source: Nat Cardiovasc Res. 2024 Jun 14;3(6):734–53. doi: 10.1038/s44161-024-00487-z (PMC11358038; doi:10.1038/s44161-024-00487-z)

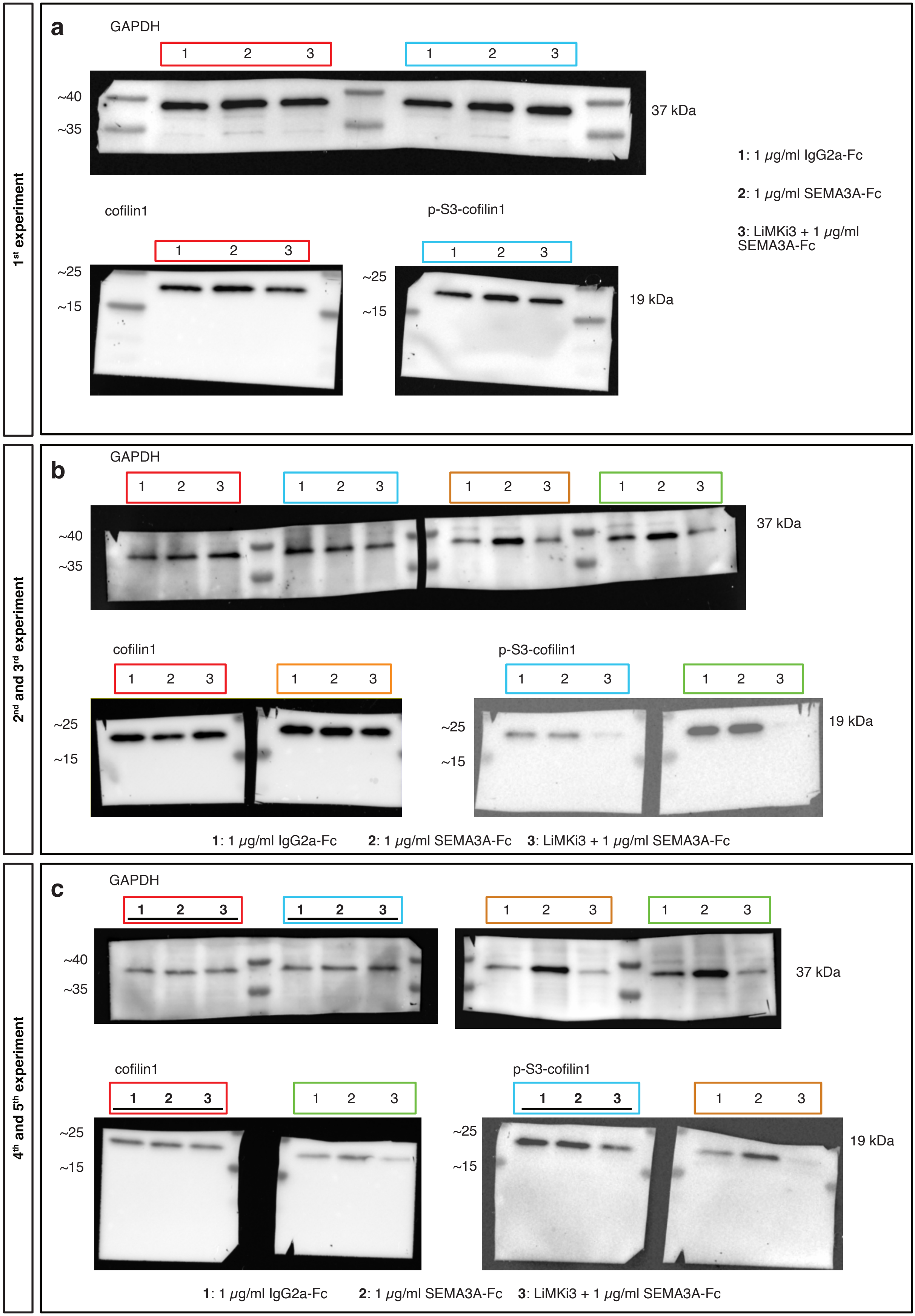

Figure 5b - Unprocessed blots

Figure 5c - SEM images

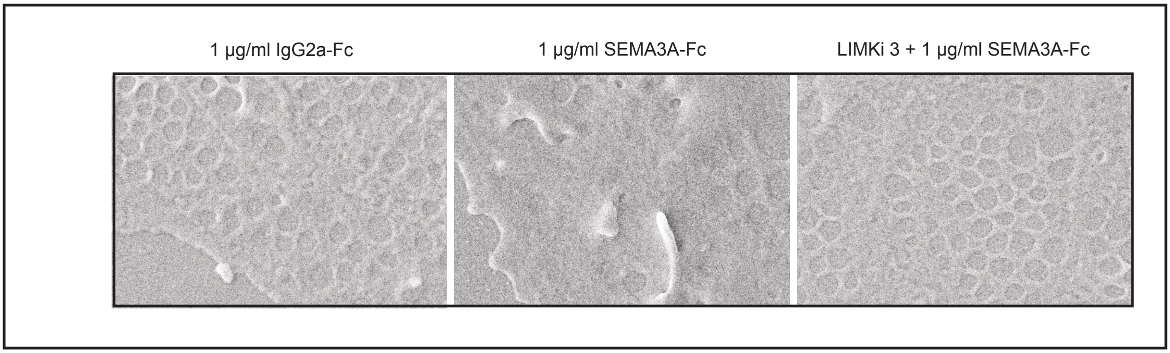

Supplement: Supplementary file 7 — Unprocessed cofilin western blots and unprocessed SEM images. [file 44161_2024_487_MOESM7_ESM.pdf]
